# Joint Association of Cholesterol, High‐Density Lipoprotein and Glucose Index, and Circadian Syndrome With Incidence of Cardiovascular Disease: Results From National Longitudinal Prospective Studies

**DOI:** 10.1155/cdr/1001613

**Published:** 2026-07-07

**Authors:** Shenghao Jiang

**Affiliations:** ^1^ School of Clinical Medicine, Faculty of Medicine & Health, UNSW Sydney, New South Wales, Australia, unsw.edu.au

**Keywords:** CHARLS, CHG, CircS, CVD, prospective cohortS

## Abstract

**Background:**

The cholesterol, high‐density lipoprotein, and glucose (CHG) index and circadian syndrome (CircS) are potential contributors for cardiovascular disease (CVD). This study evaluated their independent, combined associations, and interactions with CVD risk.

**Methods:**

The study included participants aged ≥ 45 years without baseline CVD. Participants were classified according to the median or quartile levels of the CHG index and the binary status of CircS. Univariate and multivariate Cox regression models, along with Fine–Gray competing risk models assessed individual and joint effects. Both additive and multiplicative interactions were evaluated. Restricted cubic spline (RCS) analyses were conducted to visualize the dose–response relationship between the CHG index and CVD risk. Receiver operating characteristic (ROC) curve analyses assessed the predictive performance with the SCORE2 Asia‐Pacific model for CVD at multiple time points.

**Results:**

Among 6739 eligible participants, 1420 (21.1%) participants developed CVD during follow‐up. Both higher CHG index and CircS were independently associated with increased CVD risk. Compared with low CHG index and no CircS, participants with high CHG index and CircS had higher CVD risk (hazard ratio (HR)): 1.67, 95% confidence interval (CI): 1.46–1.90). No significant additive or multiplicative interactions were observed, but the significant ones were identified in external validation ELSA cohort. Integrating CHG index and CircS modestly improved SCORE2 Asia‐Pacific model predictions, especially for 5‐, 7‐, and 9‐year long‐term CVD risk.

**Conclusions:**

Elevated CHG index and CircS independently associated with a higher risk of CVD. Significant interactions were identified, incorporating them into the SCORE2 Asia‐Pacific model modestly enhances long‐term CVD risk prediction. These readily measurable markers may therefore be applied to earlier identify high‐risk individuals, enabling more targeted CVD prevention strategies and optimize risk management in clinical practice.

**What Is Already Known**

Cardiovascular disease (CVD) represents a major global health burden with an increasing incidence worldwide. The cholesterol, high‐density lipoprotein, and glucose (CHG) index has recently emerged as a novel metabolic indicator and potential predictor of CVD risk. Circadian syndrome (CircS) encompasses a cluster of cardiometabolic disturbances contributing to CVD and diabetes. However, the quantitative contributions of the CHG index and CircS to CVD risk remain insufficiently elucidated, and their combined effect on CVD incidence has not been clearly defined.

**What Is the Key Research Question?**

What are the independent, combined associations, and interactions of CircS and CHG index with CVD risk.

**What this Study Adds**

This study demonstrates that both the CHG index and CircS independently and jointly serve as potential predictors of CVD risk. Although elevated CHG index values and the presence of CircS were each associated with a higher likelihood of developing CVD, no significant additive or multiplicative interactions were observed between them, however, significant ones were identified in English Longitudinal Study of Ageing (ELSA) validation cohort. Compared with the SCORE2 Asia‐Pacific model alone, integrating the CHG index and CircS modestly enhanced long‐term predictive performance.

**What Is the Clinical/Translational Applicability?**

This study provides prospective evidence that evaluating CHG index and CircS assessments may improve CVD risk stratification and facilitate more personalized prevention strategies for individuals at elevated cardiovascular risk.

## 1. Introduction

CVD is recognized as a major contributor to the global burden of disease, with the total patient number substantially rising from 1990 to 2019 [1]. In China, the age‐standardized incidence of CVD was estimated at approximately 411.8 per 100,000 population, whereas the corresponding mortality rate reached 245.39 per 100,000 in 2020 [2]. Moreover, CVD remains the leading reason to the worldwide disease burden, emphasizing the urgent need for more effective strategies in risk stratification, prognostic assessment, early detection, and personalized prevention and treatment [3–6]. Effective primary prevention strategies can not only reduce patient suffering but also alleviate the associated economic burden. One promising approach involves identifying robust biomarkers for CVD risk stratification [7, 8]. Although biomedical factors such as fasting blood glucose (FBG) and blood cholesterol serve as useful indicators of CVD risk, integrating large‐scale biomarkers provides a more entire evaluation and may enhance predictive accuracy compared with single‐biomarker approaches [9–11].

CircS has recently been proposed as a comprehensive clinical construct that integrates multiple cardiometabolic risk factors and associated comorbid conditions, indicating an elevated susceptibility to CVD and Type 2 diabetes (T2D) [12]. Building upon the framework of metabolic syndrome (MetS), CircS comprises central obesity, elevated fasting plasma glucose (FPG), dyslipidemia characterized by increased triglyceride (TG) levels and/or reduced high‐density lipoprotein cholesterol (HDL‐C), as well as elevated blood pressure. Notably, CircS further extends this definition by incorporating three additional domains—sleep disturbances, depressive symptoms, and nonalcoholic fatty liver disease (NAFLD) [13]. Increasing evidence indicates that disturbances in circadian timing associated with irregular sleep, mood disorders, and metabolic liver disease, which contributes to systemic metabolic dysregulation. Herein, the inclusion of sleep disturbances, depressive symptoms, and NAFLD reflects clinical manifestations closely linked to circadian misalignment. Extending beyond the conventional framework of MetS, CircS is fundamentally characterized by disturbances in circadian rhythmicity, which are governed by the hypothalamic suprachiasmatic nucleus, often termed the master circadian clock. This central regulator coordinates a wide array of physiological functions, including hormonal release, gene transcription, behavioral patterns, and energy homeostasis [13]. Therefore, CircS extends beyond the conventional MetS framework by emphasizing the role of circadian dysregulation as a unifying mechanism underlying both metabolic and neurobehavioral abnormalities. Disruption of this regulatory system leads to widespread metabolic dysregulation, representing a central mechanism in the pathogenesis of CircS [14]. Accordingly, addressing factors that contribute to circadian rhythm disturbances is crucial, as such interventions may help prevent or reverse CircS and, in turn, mitigate its associations with CVD and T2D. Recent evidence suggests that targeting circadian rhythm–related genes may help restore circadian integrity, reestablish diurnal metabolic patterns in cardiac tissue, and reduce myocardial injury and metabolic stress, which are precursors of CVD [15, 16].

Insulin resistance (IR) is a well‐established risk factor for CVD, and several indices have been developed to estimate IR, such as the triglyceride–glucose (TyG) index. However, ongoing research continues to identify novel indicators of IR [17–19]. A relatively new biomarker, the CHG index has been proposed as an effective tool for identifying T2D, and has demonstrated superior predictive performance compared with the TyG index [20]. Mansoori et al. demonstrated CHG index had better performance over TyG in predicting T2D risk (area under the receiver operating characteristic curves (AUROC): 0.864 vs. 0.825), CHG‐body mass index (CHG‐BMI) had better performance over TyG‐BMI (AUROC: 0.735 vs. 0.698), CHG‐waist circumference (CHG‐WC) index had better performance over TyG‐WC (AUROC: 0.790 vs. 0.750). Furthermore, the CHG index has shown greater predictive utility than the visceral adiposity index (VAI), lipid accumulation product (LAP), and TyG index in relation to the incidence of diabetic nephropathy (DN) and diabetic retinopathy (DR) [21].

Nevertheless, the specific role of the CHG index in predicting CVD risk remains insufficiently explored. Moreover, the longitudinal associations of both CircS and the CHG index with CVD risk, as well as their potential synergistic effects, have not been clearly established. Accordingly, leveraging longitudinal data from a large‐scale prospective cohort, the present study wanted to: [1] examine both the independent and joint associations of the CHG index and CircS with the incidence of CVD; (2) explore potential interaction effects between the CHG index and CircS; and (3) evaluate and compare the predictive utility of these measures against the established Systematic COronary Risk Evaluation 2 (SCORE2) Asia‐Pacific model.

## 2. Methods and Materials

### 2.1. Study Design and Population

This study was mainly based on prospectively collected data from the China Health and Retirement Longitudinal Study (CHARLS). Launched in 2011, CHARLS is an ongoing, nationally representative longitudinal survey targeting Chinese adults aged 45 years and older. The study adopts a multistage, stratified probability‐proportionate‐to‐size sampling strategy covering 450 communities across 28 provinces, and enrolled more than 17,000 participants at baseline [22]. Data were obtained through standardized, face‐to‐face interviews conducted by trained staff from Peking University, with five survey waves completed to date (2011–2020; Wave 1: 2011/2012, Wave 2: 2013, Wave 3: 2015, Wave 4: 2018, and Wave 5: 2020). The CHARLS protocol was approved by the Biomedical Ethics Review Committee of Peking University (IRB00001052‐11015), and written informed consent was obtained from all participants [22]. Further details regarding the study design, interview procedures, and questionnaire items are publicly available via CHARLS official website http://CHARLS.pku.edu.cn/en.

**Figure 1:**
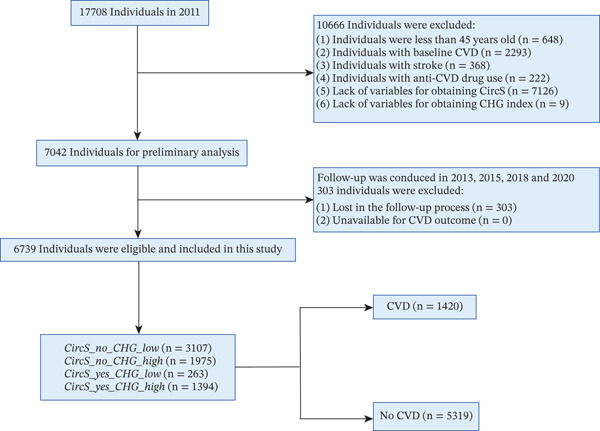
The flow chart of participants selection.

The study flowchart is presented in Figure 1. In accordance with the study protocol, data from the 2011–2020 period were analyzed. Among the 17,708 participants initially enrolled at baseline, 10,969 individuals were excluded based on the selection and exclusion criteria: age below 45 years (*n* = 648); preexisting CVD (*n* = 2293); baseline stroke (*n* = 368); current use of antiCVD medication at baseline (*n* = 222); insufficient data to determine CircS status (*n* = 7126); incomplete data to calculate the CHG index (*n* = 9); and loss to follow‐up (*n* = 303). After exclusions, a total of 6739 participants were included in the final analysis, among whom 1420 (21.1%) developed incident CVD during follow‐up. Based on the median CHG index value and CircS status, participants were categorized into four groups: CircS_no_CHG_low (*n* = 3107, 46.1%), CircS_no_CHG_high (*n* = 1975, 29.3%), CircS_yes_CHG_low (*n* = 263, 3.9%), and CircS_yes_CHG_high (*n* = 1394, 20.7%).

### 2.2. Data Imputation

Before data imputation, missing value for frailty index was around 20%, other variables had missing value less than 5%, the average missing value in total eligible participants was 0.6%. Only variables with less than 20% missingness were suitable for imputation. Overall, the data quality was satisfying and suitable for imputation. Missing data were dealt with using the Python miceforest package, which performs multiple imputation by chained equations (MICE) integrated with random forest algorithms to impute missing values, thereby effectively capturing complex nonlinear relationships. The algorithm can be found here: https://github.com/AnotherSamWilson/miceforest. The imputation procedure involved initializing missing values, iteratively refining imputations across multiple cycles, and generating several complete datasets to account for uncertainty. Specifically, 10 imputed datasets were generated, each undergoing 10 iterations to minimize stochastic variability and improve imputation accuracy. This method offers robustness in handling nonlinear data structures, accommodates both categorical and continuous variables, and enables the quantification of imputation uncertainty through multiple imputation modeling.

### 2.3. CircS Status and CHG Index Assessment

Within the CHARLS cohort, CircS status was defined according to the presence of seven components: (1) increased waist circumference (male: ≥ 85 cm, female: ≥ 80 cm); (2) elevated blood pressure, defined as systolic blood pressure (SBP) ≥ 130 mmHg and/or diastolic blood pressure (DBP) ≥ 85 mmHg, or current use of antihypertensive medication; (3) impaired FPG (≥ 100 mg/dL or use of glucose‐lowering agents); (4) elevated TG (≥ 150 mg/dL or use of TG‐lowering medication); [5] reduced HDL‐C concentrations (male: < 40 mg/dL, female: < 50 mg/dL or medication use); [6] depressive symptoms, assessed using the 10‐item Center for Epidemiologic Studies Depression Scale (CES‐D), with a score ≥ 10; and [7] short sleep duration, defined as < 6 hours per day. Participants meeting at least four of these criteria were defined as having CircS [13, 23].

Total cholesterol (TC), FPG, and HDL‐C were achieved from CHARLS cohort directly. The CHG index was calculated via this formula: CHG index = Ln [TC (mg/dL) × FBG (mg/dL)/2 × HDL − C (mg/dL)] [24, 25].

### 2.4. Assessment on Endpoint of CVD Events

In this study, the incidence of CVD was defined as the primary endpoint. CVD encompassed both heart disease and stroke, and diagnoses were estimated using standardized questions developed under the CHARLS protocol. Specifically, participants were asked whether a doctor or other healthcare professional had ever informed them of a diagnosis of heart attack, coronary heart disease, angina, congestive heart failure, or other cardiac conditions. Cerebrovascular events were ascertained through a separate question inquiring whether participants had ever been told by a healthcare professional that they had experienced a stroke. Participants were enrolled in the CHARLS cohort in 2011 and followed up in 2013, 2015, 2018, and 2020. If a participant reported a CVD event at any follow‐up prior to 2020, the CVD outcome was coded as “yes,” and the follow‐up duration for Cox regression analysis was calculated from baseline (2011) to the first reported CVD event. If a participant died without reporting a CVD event, the outcome was recorded as “no” and categorized as a “competing event,” with follow‐up time measured from 2011 to the year of death. Participants who remained alive and free of CVD through 2020 were coded as “no,” with a total follow‐up duration of 9 years.

### 2.5. Covariables

Well‐trained interviewers administered a structured questionnaire to systematically collect participant information. Covariates included sociodemographic factors: age, sex, educational level, marital status, and place of residence and health‐related characteristics, such as height, weight, smoking and drinking status, daily cigarette and alcohol consumption, eating disorders, dyslipidemia, diabetes, hypertension, obesity, angina, congestive heart failure, arrhythmia, liver disease, kidney disease, digestive disease, psychiatric disorders, cognitive impairment, cancer, fatigue, MetS, DBP, SBP, BMI, medication use for the aforementioned conditions, instrumental activities of daily living (IADL) score, and weekly exercise frequency. Additionally, laboratory and biochemical measurements included TC, TG, HDL‐C, low‐density lipoprotein cholesterol (LDL‐C), FPG, glycosylated hemoglobin A1c (HbA1c), C‐reactive protein (CRP), TyG, TyG‐BMI, and hemoglobin (HGB).

Specifically, with this formula: BMI (kg/m^2^) = body mass(kg)/height(m)^2^, we could calculate BMI. Based on self‐reported diabetes history, antidiabetic drug use, FPG ≥ 7.0 mmol/L or HbA1c levels ≥ 6.5%, diabetes was identified [26]. Diagnosis on hypertension was made by enquiring hypertension history, antihypertensive drug administration, SBP ≥ 140 mmHg, or DBP ≥ 90 mmHg [27]. With self‐reported dyslipidemia history, lipid‐reducing administration or biochemistry evaluation of G ≥ 150 mg/dL, TC ≥ 240 mg/dL, HDL‐C < 40 mg/dL, or LDL‐C ≥ 160 mg/dL, dyslipidemia was confirmed [28].

### 2.6. The SCORE2 Asia‐Pacific Model

Contemporary clinical guidelines advocate the application of robust risk prediction models to enhance CVD surveillance and inform population‐based prevention strategies. Such models synthesize multiple CVD‐related risk factors to quantify an individual′s estimated 10‐year risk of developing CVD. The SCORE2 model, introduced in the 2021 European Society of Cardiology (ESC) guidelines for CVD prevention, incorporates competing risk adjustments and systematic [29, 30]. However, as SCORE2 was primarily developed for Western populations, there is a growing need for large‐scale, region‐specific models tailored to Asian populations, given the rising CVD mortality rates and differing distributions of CVD risk factors across the Asia‐Pacific region. The SCORE2 Asia‐Pacific model was developed to address these differences, recalibrated using regional data and coefficients derived from native cohorts, and validated through extensive external validation [31]. In brief, the SCORE2 Asia‐Pacific model estimates CVD risk using variables including sex, age (≥ 50 years), current smoking status, SBP (140 mmHg), TC (5.5 mmol/L), and HDL‐C (1.3 mmol/L). In this study, I aimed to examine whether incorporating CircS and CHG index could enhance the predictive performance of the SCORE2 Asia‐Pacific model.

### 2.7. Statistical Analysis

The distributional normality of continuous variables was evaluated using the Kolmogorov–Smirnov (K–S) test, whereas the equality of variances was examined with Levene′s test. For variables demonstrating approximate normality, results were reported as means± standard deviations (SD), variables with nonnormal distributions were described using medians and interquartile ranges (IQRs). Categorical data were presented as counts along with corresponding percentages. The median CHG index value (5.27) was used to categorize participants into low and high CHG index groups. Additionally, quartile‐based classifications were established using the 25th and 75th percentiles (5.02 and 5.55, respectively), defining four categories: Q1 (2.67–5.02), Q2 (5.02–5.27), Q3 (5.27–5.55), and Q4 (5.55–8.94). Based on CircS status and CHG index levels, participants were further divided into four groups: CircS_no_CHG_low, CircS_no_CHG_high, CircS_yes_CHG_low, and CircS_yes_CHG_high. Group comparisons were conducted using the chi‐square test (or Fisher′s exact test, where appropriate) for categorical variables, and the Wilcoxon rank‐sum test, one‐way ANOVA, or Kruskal–Wallis *H* test for continuous variables, depending on data distribution characteristics.

Kaplan–Meier (K–M) survival curves were used to estimate the cumulative incidence of CVD, log‐rank test was applied to tell the differences between groups. As no violation of the proportional hazard assumption was detected, Cox proportional hazards regression models including unadjusted, crude, partially adjusted, and fully adjusted models were employed to evaluate the associations of CircS and CHG index with new‐onset CVD. Results were presented as hazard ratios (HRs) with corresponding 95% confidence intervals (CIs). To account for the competing risk of noncardiovascular death, the Nelson–Aalen estimator was applied to calculate cumulative incidence rates, and the Fine–Gray competing risk regression model was used to estimate subdistribution hazard ratios (sHRs) with 95% CIs, considering noncardiovascular deaths as competing events. Additionally, potential interactions between CircS and the CHG index were assessed on both multiplicative and additive scales. Multiplicative interaction between CircS and the CHG index was assessed by including a cross‐product term in the Cox proportional hazards model. Additive interaction was evaluated using three metrics: [1] The relative excess risk due to interaction (RERI) was employed to capture the additional risk contributed by the interaction, calculated as the difference between the observed combined effect and the sum of the individual effects of each exposure. [2] the attributable proportion due to interaction (AP) was applied to represent the share of the outcome that is attributable to the joint influence of both exposures. [3] The synergy index (SI) was determined as the ratio of the excess risk arising from simultaneous exposure to both factors relative to the cumulative excess risks associated with each factor separately.

To visually illustrate the association between the CHG index and CVD onset, restricted cubic spline (RCS) models on a continuous scale were constructed based on the Cox regression framework. Model knot selection followed the Bayesian information criterion (BIC), with four knots identified as providing the best model fit. The likelihood ratio test was applied to assess potential nonlinear relationships within the RCS models and to determine optimal cutoff points, using the “segmented” R package. The adjustment variables included in the RCS analyses were identical to those used in the corresponding Cox regression models.

To illustrate whether incorporating CircS and CHG index singly or combined into well‐established SCORE2 Asia‐Pacific model could help to improve the predictive applicability of the single model for CVD risk, I performed receiver operating characteristic (ROC) analysis [31]. The ROC curves were plotted at a 3‐year, 5‐year, 7‐year, and a 9‐year intervals to evaluate the predictive performance at these time points, the AUROC was taken to quantify the predictive performance.

Subgroup and sensitivity analysis were conducted to show robustness of current results, explore potential heterogeneity and further investigate the association. I excluded individuals with identified memory and/or psychiatric disorders, excluded cognitive impaired individuals to repeat Cox regression analysis to minimize recall biases and self‐reported biases. Then, I utilized generalized estimating equation (GEE) models to double‐validate the Cox regression model results. Deriving the advantages of estimating equations, GEE focused on population‐averaged effects and were more suitable for longitudinal data [32]. Importantly, an external cohort of ELSA (https://www.elsa-project.ac.uk/) was involved, steps and similar analyses were performed like CHARLS to test and display the robustness of findings derived from CHARLS, which could be retreated as another kind of sensitivity analyses. ELSA data and related documentations are available through the official website and the UK Data Service. Ethical approval was obtained for each wave from relevant research ethics committees. Subgroup analysis was carried out in different populations based on variables.

This study followed the Strengthening the Reporting of Observational Studies in Epidemiology (STROBE) guidelines (Table S1) [33]. All statistical analyses were performed using Python 3.9.0 (https://www.python.org/) and R 4.3.0 (https://www.r-project.org/), used packages were listed like “gtsummary,” “ggplots,” “car,” “cmprsk,” “survminer,” “rms,” “rcssci,” “geepack,” “jstable,” “timeROC,” etc. and were addressed throughout the article with statistical significance set at a two‐sided *p* value < 0.05.

## 3. Results

### 3.1. Baseline Characteristics of Included Populations

Following the selection criteria, a total of 6739 participants were included in the analysis, with a maximum follow‐up duration of 9 years. Of these, 3543 (53%) were female, and the median age was 58.0 years (IQR: 52.0–65.0). CircS was present in 1657 (25%) individuals, and the median CHG index was 5.27 (IQR: 5.02–5.55). During follow‐up, 1420 (21.1%) participants developed CVD. Detailed baseline characteristics are summarized in Table 1. In the nonCVD group, participants were distributed as follows: CircS_yes_CHG_high, 1015 (19%); CircS_yes_CHG_low, 195 (3.7%); CircS_no_CHG_high, 1551 (29%); and CircS_no_CHG_low, 2558 (48%). In the CVD group, the corresponding counts were: 379 (27%), 68 (4.8%), 424 (30%), and 549 (39%), respectively. These differences were statistically significant (*p* < 0.001), with a notably higher proportion of CircS_yes_CHG_high individuals at baseline in the CVD group. Overall, 1210 (23%) individuals in the nonCVD group and 447 (31%) in the CVD group had CircS, indicating a higher prevalence of CircS among those who developed CVD (*p* < 0.001). Additionally, participants in the CVD group exhibited more severe health conditions, including hypertension, dyslipidemia, diabetes, liver and kidney disease, higher BMI, greater prevalence of MetS, and elevated TyG and TyG‐BMI indices (all *p* < 0.001).

**Table 1:** Basic demographic characteristics of included participants.

Variables	Overall (*n* = 6739) (N (%)/mean ± SD/(Q1, Q3))	No CVD (*n* = 5319) (N (%)/mean ± SD/(Q1, Q3))	CVD (*n* = 1420) (*n* (%)/mean ± SD/(Q1, Q3))	*p*
CircS (yes)	1657 (25%)	1210 (23%)	447 (31%)	< 0.001
CHG index	5.27 (5.02, 5.55)	5.25 (5.01, 5.52)	5.33 (5.07, 5.61)	< 0.001
CHG index quantile				< 0.001
Q1	1685 (25%)	1384 (26%)	301 (21%)	
Q2	1685 (25%)	1369 (26%)	316 (22%)	
Q3	1684 (25%)	1308 (25%)	376 (26%)	
Q4	1685 (25%)	1258 (24%)	427 (30%)	
CircS_CHG_index				< 0.001
CircS_no_CHG_low	3107 (46%)	2558 (48%)	549 (39%)	
CircS_no_CHG_high	1975 (29%)	1551 (29%)	424 (30%)	
CircS_yes_CHG_low	263 (3.9%)	195 (3.7%)	68 (4.8%)	
CircS_yes_CHG_high	1394 (21%)	1015 (19%)	379 (27%)	
Gender				0.005
Female	3543 (53%)	2749 (52%)	794 (56%)	
Male	3196 (47%)	2570 (48%)	626 (44%)	
Hypertension (yes)	1484 (22%)	1017 (19%)	467 (33%)	< 0.001
Dyslipidemia (yes)	544 (8.1%)	354 (6.7%)	190 (13%)	< 0.001
Diabetes (yes)	352 (5.2%)	242 (4.5%)	110 (7.7%)	< 0.001
Cancer (yes)	46 (0.7%)	38 (0.7%)	8 (0.6%)	0.540
Lung disease (yes)	574 (8.5%)	409 (7.7%)	165 (12%)	< 0.001
Liver disease (yes)	197 (2.9%)	138 (2.6%)	59 (4.2%)	0.002
Kidney disease (yes)	321 (4.8%)	238 (4.5%)	83 (5.8%)	0.031
Gastric disease (yes)	1400 (21%)	1051 (20%)	349 (25%)	< 0.001
Psychiatric disorder (yes)	63 (0.9%)	44 (0.8%)	19 (1.3%)	0.076
Memory related disorder (yes)	66 (1.0%)	49 (0.9%)	17 (1.2%)	0.350
Arthritis (yes)	2185 (32%)	1662 (31%)	523 (37%)	< 0.001
Asthma (yes)	248 (3.7%)	163 (3.1%)	85 (6.0%)	< 0.001
Residential area				0.710
Rural	4428 (66%)	3489 (66%)	939 (66%)	
Urban	2311 (34%)	1830 (34%)	481 (34%)	
Marriage				0.370
Married and living with spouse	5693 (84%)	4490 (84%)	1203 (85%)	
Not married	47 (0.7%)	41 (0.8%)	6 (0.4%)	
Others	999 (15%)	788 (15%)	211 (15%)	
Drinking	2329 (35%)	1879 (35%)	450 (32%)	0.010
Smoking	2109 (31%)	1700 (32%)	409 (29%)	0.023
Age, year	58.00 (52.00, 65.00)	57.00 (51.00, 64.00)	59.00 (54.00, 66.00)	< 0.001
Education				0.056
Below primary school	3396 (50%)	2658 (50%)	738 (52%)	
High school and above	648 (9.6%)	508 (9.6%)	140 (9.9%)	
Middle school	1272 (19%)	1040 (20%)	232 (16%)	
Primary school	1423 (21%)	1113 (21%)	310 (22%)	
Normal physical activity (yes)	6307 (94%)	4989 (94%)	1318 (93%)	0.180
DBP, mmhg	74.00 (66.50, 82.50)	73.50 (66.00, 82.00)	76.50 (68.50, 85.00)	< 0.001
SBP, mmhg	125.50 (113.00, 140.50)	124.50 (112.50, 139.00)	130.00 (117.00, 145.50)	< 0.001
Frailty index	10.60 (5.58, 16.74)	10.01 (5.13, 14.88)	11.82 (7.81, 18.88)	< 0.001
BMI, kg/m2	23.05 (20.81, 25.62)	22.90 (20.69, 25.36)	23.80 (21.30, 26.62)	< 0.001
MetS	3926 (58%)	2961 (56%)	965 (68%)	< 0.001
Height, cm	1.58 (1.52, 1.64)	1.58 (1.52, 1.64)	1.58 (1.52, 1.64)	0.840
Waist, cm	84.00 (77.20, 91.30)	83.40 (77.00, 90.50)	87.00 (79.20, 94.20)	< 0.001
Weight, kg	57.50 (50.80, 65.40)	57.10 (50.50, 64.80)	59.40 (52.20, 67.80)	< 0.001
Pulse	71.50 (65.00, 78.50)	71.50 (65.00, 78.00)	72.00 (65.50, 79.00)	0.008
Antidiabetes drug use	219 (3.2%)	145 (2.7%)	74 (5.2%)	< 0.001
Antidyslipidemia drug use	274 (4.1%)	177 (3.3%)	97 (6.8%)	< 0.001
Antihypertension drug use	1043 (15%)	707 (13%)	336 (24%)	< 0.001
Antikidney drug use	189 (2.8%)	138 (2.6%)	51 (3.6%)	0.043
Antiliver disease drug use	111 (1.6%)	81 (1.5%)	30 (2.1%)	0.120
Antilung disease drug use	389 (5.8%)	270 (5.1%)	119 (8.4%)	< 0.001
Antimemory related disorder drug use	29 (0.4%)	19 (0.4%)	10 (0.7%)	0.076
Antipsychiatric disorder drug use	37 (0.5%)	29 (0.5%)	8 (0.6%)	0.930
Psychiatric disorder treatment	13 (0.2%)	7 (0.1%)	6 (0.4%)	0.038
TyG	8.56 (8.20, 9.00)	8.54 (8.18, 8.98)	8.65 (8.29, 9.07)	< 0.001
TyG BMI	197.93 (174.69, 226.55)	195.88 (172.93, 223.10)	207.77 (179.87, 236.74)	< 0.001
Glu, mg/dL	102.06 (94.32, 111.78)	101.70 (94.14, 111.24)	102.96 (94.86, 113.94)	< 0.001
TC, mg/dL	190.98 (167.78, 215.72)	190.59 (167.40, 214.95)	193.30 (170.30, 219.59)	0.003
TG, mg/dL	102.66 (73.46, 148.68)	100.00 (71.68, 146.02)	108.86 (78.76, 155.76)	< 0.001
HDL‐C, mg/dL	49.87 (40.98, 60.70)	50.26 (40.98, 61.08)	48.71 (40.40, 58.57)	< 0.001
LDL‐C, mg/dL	115.21 (93.94, 138.02)	114.43 (93.56, 136.86)	116.95 (96.26, 141.11)	0.002
CRP, mg/dL	1.00 (0.54, 2.09)	0.95 (0.53, 1.98)	1.17 (0.59, 2.32)	< 0.001
HbA1c, %	5.10 (4.90, 5.40)	5.10 (4.90, 5.40)	5.20 (4.90, 5.50)	< 0.001
HGB, g/dL	14.20 (13.00, 15.50)	14.20 (13.00, 15.50)	14.30 (13.10, 15.60)	0.280
Cognitive status (yes)	1710 (25%)	1369 (26%)	341 (24%)	0.180

*Note:* Values are presented in N (%) or mean ± SD, or median (Quartile 1, Quartile 3).

Abbreviations: BMI, body mass index; CHG index, cholesterol, high‐density lipoprotein and glucose (CHG) index; CircS: circadian syndrome; CRP, C‐reactive protein; CVD, cardiovascular disease; DBP, diastolic blood pressure; Glu, glucose; HDL, high‐density lipoprotein; HGB, hemoglobin; LDL, low‐density lipoprotein; MetS, metabolic syndrome; SBP, systolic blood pressure; TC, total cholesterol; TG, triglycerides; TyG, triglyceride–glucose index.

### 3.2. Association Between CircS, CHG Index and CVD

**Figure 2:**
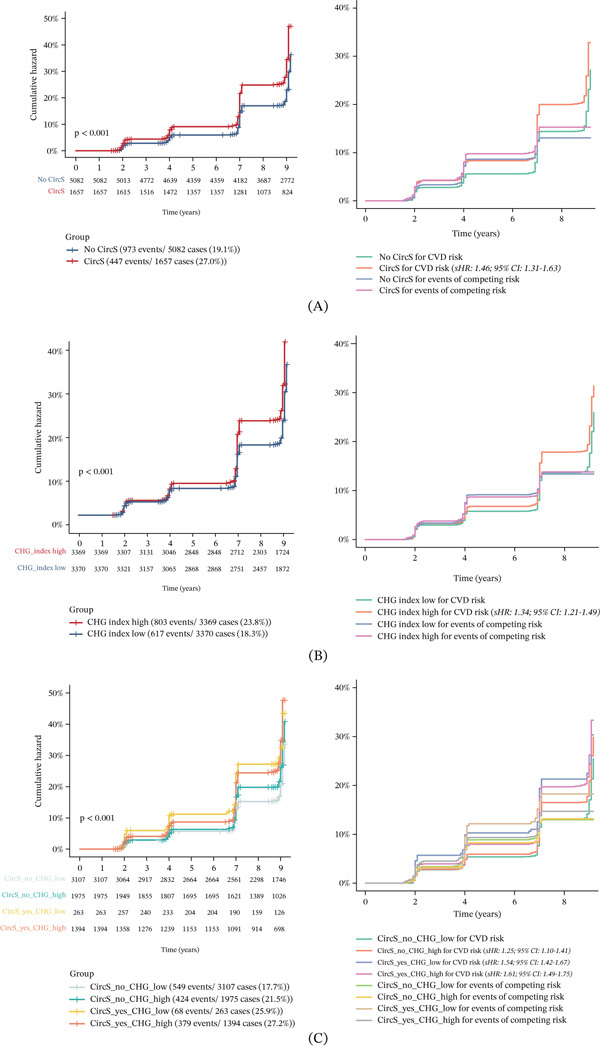
Kaplan–Meier plots for cumulative CVD risk and Fine–Gray competing risk model results. (A) Cumulative risk and Fine–Gray competing risk model results by CircS. (B) Cumulative risk and Fine–Gray competing risk model results by CHG index (classified by the median value). (C) Cumulative risk and Fine–Gray competing risk model results by the combination of CircS and CHG index. Abbreviations: CHG, cholesterol, high‐density lipoprotein and glucose; CI, confidence interval; CircS, circadian syndrome; CVD, cardiovascular disease; sHR, subdistributon hazard ratio.

Compared with participants without CircS, those with CircS exhibited a significantly higher cumulative risk of CVD during follow‐up (27.0% vs. 19.1%; *p* < 0.001), with a pronounced divergence emerging after year two, as illustrated in the K–M curve (Figure 2A). Similarly, participants in the high CHG index group experienced more CVD events than those in the low CHG index group (23.8% vs. 18.3%; *p* < 0.001), with the difference becoming prominent after year four (Figure 2B). Based on CHG index quartiles, cumulative CVD risk was highest in the Q4 group (23.3%) compared with Q3 (22.3%), Q2 (18.8%), and Q1 (17.9%; *p* < 0.001) (Figure S2). A CHG index of 5.29 was the default cutoff value by survminer package, the high CHG index group based on this cutoff value also demonstrated more CVD events (HR: 1.36, 95% CI: 1.23–1.51; *p* < 0.001). Participants in the CircS_yes_CHG_high group demonstrated the highest cumulative CVD risk compared with the other three groups (27.2% vs. 25.9% vs. 21.5% vs. 17.7%; *p* < 0.001) over the 9‐year follow‐up. Although the CircS_yes_CHG_low group maintained a leading cumulative risk from Year 2 to 8, it was surpassed by the CircS_yes_CHG_high group near Year 9, reflecting a higher number of incident CVD events in the final follow‐up year (Figure 2C). Results from the Fine–Gray competing risk model were consistent, indicating that these findings were robust and not materially affected by potential competing risks. Specifically, the sHR for CircS was 1.46 (95% CI: 1.31–1.63) (Figure 2A), for the high CHG index group 1.34 (95% CI: 1.21–1.49) (Figure 2B), and for the combined groups, CircS_yes_CHG_high: 1.61 (95% CI: 1.49–1.75), CircS_yes_CHG_low: 1.54 (95% CI: 1.42–1.67), and CircS_no_CHG_high: 1.25 (95% CI: 1.10–1.41) (Figure 2C).

Cox regression analysis results whether adjusted or not were reported in Table 2. Model 1 was adjusted for age and sex. Model 2 included all covariates in Model 1, with additional adjustment for smoking status, alcohol consumption, marital status, education level, residential area, and regular physical activity. Model 3 further accounted for clinical factors, including diabetes, hypertension, dyslipidemia, kidney disease, liver disease, and CRP, in addition to the covariates in Model 2, consistent with variable selection strategies employed in previous studies. Across all models, results were consistent with the unadjusted analyses and aligned with trends observed in the K–M plots, indicating that both CircS and a higher CHG index were independently associated with increased CVD risk. Participants with combined CircS and high CHG index exhibited the highest risk compared with those with either factor alone or neither. Notably, in Model 3, the association for the CircS_yes_CHG_low group compared with the CircS_no_CHG_low reference group was no longer statistically significant (*p* = 0.154), potentially due to the small sample size (*n* = 263) in this subgroup. Nevertheless, the HR remained elevated (HR: 1.26, 95% CI: 1.09–1.45), suggesting a trend toward increased risk.

**Table 2:** Cox regression model results for CircS and CHG index and their joint risk for CVD.

Variables	Case	Unadjusted model		Model 1		Model 2		Model 3	
		HR (95% CI)	*p*	HR (95% CI)	*p*	HR (95% CI)	*p*	HR (95% CI)	*p*
CircS									
No	5082	1 (Ref.)		1 (Ref.)		1 (Ref.)		1 (Ref.)	
Yes	1657	1.51 (1.35–1.69)	< 0.001	1.45 (1.30–1.62)	< 0.001	1.46 (1.30–1.63)	< 0.001	1.14 (1.01–1.29)	0.037
CHG index (continuous)		1.41 (1.26–1.57)	< 0.001	1.42 (1.27–1.58)	< 0.001	1.40 (1.26–1.57)	< 0.001	1.21 (1.07–1.36)	0.002
CHG index (median)									
Low	3370	1 (Ref.)		1 (Ref.)		1 (Ref.)		1 (Ref.)	
High	3369	1.36 (1.23–1.51)	< 0.001	1.36 (1.23–1.51)	< 0.001	1.35 (1.22–1.51)	< 0.001	1.22 (1.09–1.36)	< 0.001
CHG index (quartile)									
Q1	1685	1 (Ref.)		1 (Ref.)		1 (Ref.)		1 (Ref.)	
Q2	1685	1.01 (0.87–1.19)	0.856	1.00 (0.85–1.17)	0.997	0.99 (0.85–1.16)	0.918	0.97 (0.82–1.13)	0.665
Q3	1684	1.26 (1.08–1.47)	0.003	1.25 (1.08–1.46)	0.004	1.25 (1.07–1.45)	0.004	1.16 (0.99–1.35)	0.064
Q4	1685	1.49 (1.28–1.72)	< 0.001	1.47 (1.27–1.71)	< 0.001	1.45 (1.25–1.69)	< 0.001	1.24 (1.06–1.44)	0.007
CircS and CHG index									
CircS_no_CHG_low	3107	1 (Ref.)		1 (Ref.)		1 (Ref.)		1 (Ref.)	
CircS_no_CHG_high	1975	1.26 (1.11–1.43)	< 0.001	1.27 (1.12–1.45)	< 0.001	1.26 (1.11–1.43)	< 0.001	1.23 (1.08–1.40)	0.001
CircS_yes_CHG_low	263	1.65 (1.28–2.12)	< 0.001	1.52 (1.18–1.96)	0.001	1.54 (1.19–1.98)	< 0.001	1.21 (0.93–1.56)	0.154
CircS_yes_CHG_high	1394	1.67 (1.46–1.90)	< 0.001	1.61 (1.42–1.84)	< 0.001	1.62 (1.42–1.84)	< 0.001	1.26 (1.09–1.45)	0.002

*Note:* Model 1, adjusted for age and gender; Model 2, adjusted for Model 1 plus smoking, drinking, marriage, education, residential area, and normal physical activity; Model 3, adjusted for Model 2 plus diabetes, hypertension, dyslipidemia, kidney disease, liver disease, and C‐reactive protein.

Abbreviations: 95% CI, 95% confidence interval; CHG index, cholesterol, high‐density lipoprotein and glucose (CHG) index; CircS, circadian syndrome; HR, hazard ratio.

### 3.3. Interaction Between CircS and CHG Index

Investigating the synergic role and interaction in CircS and CHG index, I observed that the CIs for RERI and AP included 0, whereas those for the SI and multiplicative effects included 1 across the overall cohort and all subpopulations (Table 3). Although participants with both CircS and a high CHG index exhibited a trend toward higher CVD risk compared with either factor alone, formal statistical testing revealed no significant additive or multiplicative interactions between CircS and the CHG index in CHARLS study. This may reflect overlapping biological pathways, as both CircS and the CHG index primarily influence CVD risk through metabolic mechanisms. Additionally, more precise and validated cutoff values for the CHG index beyond the median split may be required to accurately classify high versus low levels in future studies. Beyond investigating these synergic roles in other external validation cohorts, such refinement would enable a more rigorous investigation of potential interactions across the full population and in targeted subgroups.

**Table 3:** Joint association and interactions between CircS and CHG on CVD.

Interactive indices	Interactive effects (95% CI)															
	In all populations (95% CI)	*p*	Male (95% CI)	*p*	Female (95% CI)	*p*	Individuals with hypertension (95% CI)	*p*	Individuals with dyslipidemia (95% CI)	*p*	Individuals with diabetes (95% CI)	*p*	Individuals with liver disease (95% CI)	*p*	Individuals with kidney disease (95% CI)	*p*
Additive effect																
RERI	−0.24 (−0.70, 0.21)	0.851	0.07 (−0.58, 0.72)	0.420	−0.48 (−1.11, 0.15)	0.932	−0.22 (−0.74, 0.31)	0.788	−0.38 (−2.64, 1.87)	0.630	−0.38 (−2.64, 1.87)	0.630	−0.04 (−1.60, 1.51)	0.522	−1.30 (−3.83, 1.22)	0.845
AP	−0.14 (−0.42, 0.13)	0.152	−0.04 (−0.34, 0.42)	0.420	−0.29 (−0.69, 0.10)	0.072	−0.18 (−0.63, 0.26)	0.211	−0.16 (−1.09, 0.76)	0.364	−0.16 (−1.09, 0.76)	0.364	−0.03 (−1.12, 1.06)	0.478	−0.69 (−2.08, 0.69)	0.164
SI	0.73 (0.44, 1.24)	0.878	1.10 (0.41, 2.99)	0.423	0.56 (0.30, 1.05)	0.965	0.44 (0.10, 2.04)	0.852	0.78 (0.23, 2.67)	0.655	0.78 (0.23, 2.67)	0.655	0.91 (0.03, 24.64)	0.523	0.40 (0.11, 1.52)	0.910
Multiplicative scale	0.80 (0.60, 1.07)	0.134	0.98 (0.61, 1.57)	0.935	0.70 (0.48, 1.01)	0.056	0.82 (0.52, 1.29)	0.401	0.69 (0.20, 2.33)	0.547	0.69 (0.20, 2.33)	0.547	0.94 (0.26, 3.35)	0.921	0.47 (0.11, 1.52)	0.163

Abbreviations: AP, proportion attributable to interaction; CI, confidence interval; RERI, relative excess risk due to interaction; SI, synergy index.

### 3.4. RCS to Visualize Relationship Between CHG Index and CVD Risk

**Figure 3:**
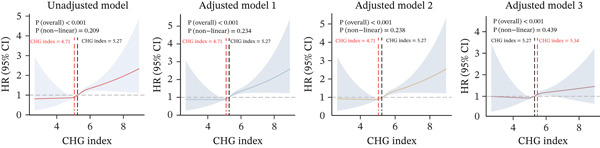
RCS models for the relationship between CHG index and CVD risk. Abbreviations: CHG, cholesterol, high‐density lipoprotein and glucose; CI, confidence interval; CVD, cardiovascular disease; HR, hazard ratio; RCS, restricted cubic spline. Covariables used for adjusting for different models were addressed in the main text.

Based on previous analyses, the CHG index appeared to exhibit a positive “dose‐response” relationship with CVD risk. To visualize this association, RCS models were fitted within both unadjusted and adjusted Cox regression frameworks. The RCS analyses demonstrated a positive linear relationship between the CHG index and CVD risk, with no evidence of significant nonlinearity (*p* for overall < 0.001; *p* for nonlinear = 0.209). This linear trend persisted after adjustment for covariates (Figure 3). Inflection point and cutoff point were identified, respectively, in the spline models with RCS and segmented regression model. The first inflection point, indicated by a red dotted line, represented a point at which the relationship exhibits distinct trends on either side. The second cutoff point (also known as reference point, defaulted set as the median value of CHG index by RCS), marked by a black dotted line, corresponded to the point where the HR equals 1.0; beyond CHG index of 5.27, increasing CHG index values are associated with progressively higher CVD risk. Specifically, when the CHG index exceeded 5.27, the risk of CVD became pronounced, underscoring the importance of timely and targeted interventions to prevent CVD. Both cutoff point and inflection point provided a practical framework for understanding the dose‐response relationship and identifying the optimal timing for preventive strategies. The absence of significant nonlinearity further supported the robustness and good fit of the Cox regression model and reinforces the consistently positive association between the CHG index and CVD risk.

### 3.5. Sensitivity and Subgroup Analysis

When restricted to individuals without memory or psychiatric disorders, without cognitive diseases, the CircS_yes_CHG_high group continued to exhibit significantly higher CVD risk compared with the CircS_no_CHG_low group across both unadjusted and adjusted models (Table S2). To complement the Cox regression analyses and address their limitations, GEE model was employed. GEE an extension of the generalized linear model (GLM), accommodates correlations among observations, such as repeated measurements within the same individual over time or data clustered within groups. By incorporating a working correlation structure, GEE effectively models relationships among correlated observations and provides robust estimates of population‐averaged effects in longitudinal data. Its advantages include flexibility in handling diverse data types, robust results to misspecified correlation structures, and computational efficiency. Using the GEE framework, the findings remained consistent, reinforcing the robustness of the observed associations between CircS, CHG index, and CVD risk.

Besides, the established relationship between CircS, CHG index and CVD risk was not significantly modified by stratified factors like age, sex, smoking, drinking, residential area, diabetes, hypertension, dyslipidemia, kidney disease, BMI, and CRP through subgroup analysis (all *p* > 0.05 for interaction) (Table S3). These findings underscore the consistency, robustness, and generalizability of the observed relationships across diverse population subgroups.

### 3.6. External ELSA Cohort Results

The ELSA cohort is a nationally representative prospective cohort study conducted on community‐dwelling adults aged 50 years and older in England. Original ELSA samples were recruited from participants in the Health Survey for England, with 12,099 individuals included in Wave 1. Since its launch in 2002, ELSA has collected repeated data approximately every 2 years, covering 11 main waves up to 2023/2024. ELSA collects comprehensive information on demographic characteristics, socioeconomic position, health behaviors, physical and mental health, cognitive function, social participation, and biomarkers in specific waves. In short, ELSA is an important resource for investigating ageing, chronic disease, disability, cognition, and social determinants of health in older adults with longitudinal manner.

To extensively satisfy accurate extraction of CircS, CHG index and both of them as well as CVD status in most of ELSA populations, I selected Wave 4 (2008) to Wave 10 (2021–2023) data for my validation analysis. After similar selection and exclusion criteria process and imputing, the utmost lack data for each covariable were no more than 8% suggesting the high quality of the covariables in included ELSA populations. Then a total of 2302 eligible individuals entered my ELSA analysis, the selection process could be found in Figure S2, distributions of key covariables between CVD (*n* = 652) and nonCVD (*n* = 1650) populations in ELSA were quite similar to CHARLS cohort (Table S4). From ELSA results, I noticed individuals with CircS, with higher CHG index and with both CircS and higher CHG index would harbor more probability to develop CVD events (Figure S3), after multicovariables adjustment, above findings kept existing, although the positive association between CHG index and CVD became not so strong in adjustment model 2 and model 3 (Table S5). Besides, in CHARLS cohort, no significant additive and multiplicative effects were identified between CircS and CHG index on CVD, though they demonstrated strong trend. However, in ELSA study, the CircS and CHG index showed significant and obvious additive and multiplicative effects on CVD events (Table S6). This finding indicated that the coexistence of CircS and a high CHG index was associated with a markedly elevated risk of CVD compared with either factor alone, highlighting the importance of implementing combined interventions rather than targeting each factor in isolation. In the RCS for CHG index, populations in ELSA exhibited similar trend like it in CHARLS study (Figure S4). Overall, as part of the sensitivity analyses, the external ELSA cohort not only validated the findings from the primary CHARLS study but also provided further evidence of both additive and multiplicative interactions between CircS and the CHG index in relation to CVD risk.

### 3.7. Predictive Performance of CircS and CHG Index

**Figure 4:**
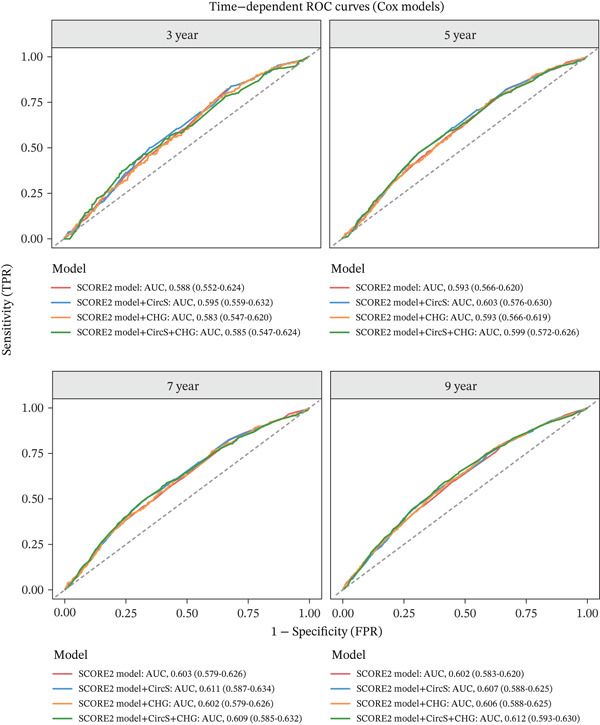
ROC curves and AUROCs (95% CI) compared the predictive efficacy of SCORE2 Asia‐Pacific model. Compared to SCORE2 Asia‐Pacific model alone, SCORE2 Asia‐Pacific model with single CircS, SCORE2 Asia‐Pacific model with single CHG index, and with the combination of CircS and CHG index for the 3‐year, 5‐year, 7‐year and 9‐year CVD risk, respectively. Abbreviations: AUROC, area under the receiver operating characteristic curve; CHG, cholesterol, high‐density lipoprotein and glucose; CI, confidence interval; CircS, circadian syndrome; CVD, cardiovascular disease; ROC, receiver operating characteristic; SCORE2, Systematic COronary Risk Evaluation 2.

ROC analyses at multiple time points were performed to evaluate the predictive performance of the SCORE2 Asia‐Pacific model with and without the inclusion of CircS, the CHG index, or their combination for CVD risk. Incorporating both CircS and CHG index into the SCORE2 Asia‐Pacific model modestly improved its predictive performance compared with the model alone at 5 years (AUROC: 0.599 (0.572–0.626) vs. 0.593 (0.566–0.620)), 7 years (AUROC: 0.609 (0.585–0.632) vs. 0.603 (0.579–0.626)), and 9 years (AUROC: 0.612 (0.593–0.630) vs. 0.602 (0.583–0.620)) (Figure 4). Despite its endorsement by the ESC, the SCORE2 Asia‐Pacific model demonstrated lower‐than‐expected performance in the CHARLS cohort. Several factors may contribute to this discrepancy: [1] CHARLS is a China‐specific cohort with unique CVD risk profiles and parameter distributions, whereas SCORE2 Asia‐Pacific is calibrated for broader regional applicability; [2] the SCORE2 Asia‐Pacific model includes both CVD and nonCVD individuals without specific restrictions on age, disease history, medications, or interventions, whereas this study applied stringent selection criteria and did not recalibrate the model using local Asian cohort parameters; [3] CHARLS data were collected from 2011–2020, whereas SCORE2 Asia‐Pacific was updated in 2022, during which advances in CVD diagnosis, prevention, and treatment may have affected predictive performance. Nonetheless, current findings indicate that adding CircS and the CHG index slightly enhances the predictive accuracy of the SCORE2 Asia‐Pacific model, particularly for long‐term CVD risk assessment.

## 4. Discussion

CVD is one of the leading causes of death globally and one of the most fatal diseases in both the US and China. In this study, I demonstrated that: [1] CircS and the CHG index are independent risk factors for incident CVD, both before and after extensive covariate adjustment; [2] competing risk events did not materially affect these associations; [3] individuals with both CircS and a high CHG index exhibited higher CVD risk, given significant additive and multiplicative interactions reached; [4] a CHG index exceeding 5.27 marked a threshold beyond which CVD risk increased progressively; and [5] integrating CircS and the CHG index into the SCORE2 Asia‐Pacific model modestly improved predictive performance at 5‐, 7‐, and 9‐year intervals compared with the model alone. Collectively, these findings underscore the value of CircS and the CHG index as novel CVD risk biomarkers, which can complement the SCORE2 Asia‐Pacific model and inform strategies for enhanced risk stratification, prognostic assessment, and targeted primary prevention of CVD.

### 4.1. Compared With Other Studies

Numerous studies have established that a high TyG index serves as a surrogate marker associated with elevated T2D and CVD risk. For instance, a comprehensive study of 403,335 participants from the UK Biobank reported a positive correlation between TyG index and CVD risk [34], although a negative nonlinear relationship emerged in the fully adjusted model after accounting for all covariates. In contrast, a study based on the CHARLS cohort using RCS identified an inverted U‐shaped nonlinear association between TyG index and CVD risk [35]. These discrepancies may reflect differences in cohort design, sample size, demographic characteristics, selection criteria, and the covariates included in adjustments. Such variability underscores the biological complexity of the relationship, which is influenced by multiple health‐related and socioeconomic factors, and highlights the need to identify precise subpopulations and alternative surrogate markers to complement or refine TyG‐based risk assessment. Similar indices, including TyG variability [36], TyG‐BMI [37], TyG‐WHR [38], TyG‐WC index [39] were always found to be associated with increased CVD risk. Moreover, a higher TyG index has been linked to stroke outcomes, including elevated inhospital mortality, reduced long‐term survival, and increased risk of stroke recurrence, further supporting its relevance as a CVD risk marker [35, 40, 41]. Investigators keep looking for new surrogates offering direct measurement, greater analytical stability, and lower cost, which aligns with the principle of higher simplicity and economic feasibility for clinical practice.

Unlike previous studies, I investigated the novel CHG index and its association with CVD risk. First introduced by Mansoori et al. [24], the CHG index demonstrated superior performance in diagnosing T2D compared with the TyG index and related variants; however, its relationship with CVD risk has not been fully elucidated. In the present study, fully adjusted Cox regression models confirmed that the CHG index is an independent risk factor for incident CVD. Importantly, I applied the Fine–Gray competing risk model for the first time, demonstrating that the CHG index′s association with CVD risk was not materially affected by competing events. Both current study and Mo et al. [25] for dynamic changes of CHG index and CVD outcome, while our study provide additional information like [1] the identification of a cut‐off point splitting the RCS into two segments with distinct trends, despite the nonlinear component being statistically insignificant; and [2] the determination of a threshold at which the HR exceeded 1.0, indicating a markedly increased CVD risk as CHG index continued to rise. These findings help define the optimal time window for primary CVD prevention by targeting the CHG index. Both of current study and Mo et al. [25] demonstrated a strong linear dose‐response relationship, although minor differences in the overall trend and CIs from the spline origin to the HR = 1 threshold likely reflect variations in participant characteristics and covariate adjustments. Similarly, Guo et al. [42] reported RCS analyses of the CHG index for CVD and all‐cause mortality, but their study was limited by a single‐center design, smaller sample size, and reduced generalizability. Furthermore, Fine–Gray analyses were not performed in Guo et al., leaving the potential influence of comorbidities, such as cancer, in CVD patients unaccounted for [42].

Beyond single CHG index, I explored the joint association and interactions between CHG index and CircS in CVD, which was never discussed before. In contrast to the CHG index, CircS is more comprehensively constructed, incorporating MetS as well as its comorbidities. Disruptions in circadian rhythms have been implicated as potential drivers of a range of chronic conditions, including cardiovascular, cerebrovascular, and renal disorders. The circadian clock modulates cardiac functions, including endothelial function, thrombosis process, heart rate, and blood pressure control [43, 44]. Manifestation of acute myocardial infraction, stroke, arrhythmia and other adverse CVD events also associates with circadian patterns. In longitudinal studies, CircS independently links to the enhanced risk for all‐cause mortality and functional ability. Zheng et al. [45] involved CHARLS and NHANES data observing linear dose‐response relationships between mortality risk and the number of CircS components, addressing the inhibition of circadian disruptions in public health. Huang et al. [46] showed suffering CircS associated with body functional ability. Based on Chinese populations, subsequent studies explored the association between CircS with cognitive impairment [47], kidney disease [48] and hyperuricemia [49] and results were consistent with studies conducted in the US populations [50, 51].

Shi et al. [13] demonstrated using CHARLS data that CircS outperformed MetS in predicting CVD incidence, and the coexistence of CircS and MetS was associated with a two‐fold increase in CVD risk. However, they did not conduct additive or multiplicative interaction analyses to formally assess statistical interactions between CircS and MetS. Notably, even when elevated HRs were observed for participants with both CircS and MetS in Cox regression models, additive or multiplicative interactions may still be nonsignificant, similar to the the CHARLS findings in the present study. Shi et al. [13] were therefore recommended to use metrics such as RERI, AP, SI, and multiplicative interaction terms to reevaluate their results and draw more cautious conclusions. They also generated ROC curves to assess the predictive efficacy of CircS, with AUROCs comparable to those in our study, highlighting the modest but potential predictive value of CircS in Chinese population‐based cohorts [13]. Although the CircS_yes_CHG_high group exhibited the highest risk of CVD, formal tests for additive and multiplicative interactions were not statistically significant in the CHARLS analysis results. This finding did not totally exclude the possibility of biological interplay between CircS and the CHG index. Statistical interaction tests are known to have relatively low power, particularly when evaluating joint effects across subgroups [52, 53]. What is more, the significant results were identified in ELSA cohort. In the present study, the increased hazard observed in individuals with both CircS and high CHG index suggested that the coexistence of these metabolic disorders confer the greatest cardiovascular risk. However, the magnitude of the combined effect did not significantly exceed the expected risk derived from their independent contributions. These results therefore indicated a cumulative or overlapping risk pattern rather than a statistically detectable synergistic interaction. Future studies with larger sample sizes or mechanistic investigations can help further clarify the potential biological interplay between circadian dysregulation and glucose homeostasis in the development of CVD. Overall, the current study represents the first longitudinal investigation examining CircS, the CHG index, and their joint interactions in relation to CVD. These stable findings provide valuable insights for real‐world CVD risk stratification and preventive strategies.

Although the addition of CircS and the CHG index resulted in an improvement in AUROC at the 9‐year interval, the absolute gain in discrimination was relatively modest. This finding suggested that while these indices might provide incremental prognostic performance beyond the SCORE2 Asia‐Pacific model, their clinical utility should be interpreted with caution. Importantly, both CircS and the CHG index are calculated from routinely available clinical parameters, which allow their integration into risk assessment without requiring additional laboratory testing. Nevertheless, whether such modest improvements in long‐term risk prediction justify their routine clinical implementation remains uncertain and warrants further validation in external cohorts as well as evaluation using additional metrics of clinical utility.

### 4.2. Biological Relevance and Clinical Application

The concept of CircS is based on the observation that multiple chronic conditions including obesity, hypertension, dyslipidemia, T2D, depression, sleep disturbances, and NAFLD [23]. Contemporary lifestyle factors, such as sleep deprivation, high‐calorie dietary intake, shift work, and exposure to artificial light can perturb circadian rhythms, consequently contributing to a wide range of adverse health outcomes [13, 23]. It has been established that disruptions in sleep patterns and the presence of depressive symptoms adversely affect circadian regulation of multiple physiological processes, thereby elevating the risk of CVD. Basically, CircS should be treated as a risk concern for CVD given the intertwined relationship with metabolic disorders. Circadian rhythm is also thought to be associated with cerebral perfusion, Philipp et al. [54] highlighted that the interplay between chronobiology and cerebrovascular diseases involves processes such as cell death, metabolic regulation, mitochondrial function, and immune/inflammatory responses. CircS not only contributes to stroke risk but also has implications for clinical outcomes [55]. Circadian regulation of activity and food intake is, in part, mediated by the kidney via intrinsic molecular mechanisms that continuously control glomerular and tubular functions, with diurnal variations observed in glomerular filtration rate (GFR), renal plasma flow (RPF), and the excretion of major urinary solutes [56]. Based on that, CircS could associate with not only CVD, stroke, but also kidney‐related diseases and cardiovascular mortality. Conversely, increased fat accumulation, reduced lean mass, and IR have been associated with CircS, collectively contributing to obesity and related disorders for example CVD [57].

As a new and comprehensive concept, CHG index may be mainly related to factors such as glucose metabolism, lipid metabolism, IR, inflammatory response, oxidative stress, and other mechanism. Endothelial dysfunction may be promoted by IR, with the resulting imbalance in the production and release of vasodilatory and vasoconstrictive factors. These factors are known to promote atherosclerosis, impairing aortic valve and vascular structure and function, and contribute to the risk of adverse outcomes [58]. Inflammation plays a central role in both the initiation and progression of aortic valve stenosis. Macrophages engulf cholesterol crystals, which leads to the formation of foam cells. When these cells rupture, they release proinflammatory mediators that attract additional immune cells, thereby sustaining a chronic inflammatory state. Elevated levels of cholesterol and glucose have been associated with worsening aortic valve lesions and a higher likelihood of CVD [25, 42, 59, 60]. Moreover, increased oxidative stress generates excessive reactive oxygen species, which can damage cellular biomolecules and promote proliferation and phenotypic changes in valvular interstitial cells, ultimately resulting in valve thickening, rigidity, and calcification.

Furthermore, IR contributes to metabolic disturbances, including hyperglycemia, dyslipidemia, and hypertension, thereby increasing cardiac workload and promoting myocardial hypertrophy, fibrosis, and ultimately CVD. Elevated cholesterol and glucose levels can alter hemorheological properties, facilitating platelet aggregation and thrombosis. Hemodynamic alterations associated with aortic valve stenosis further enhance thrombotic risk, whereas endothelial dysfunction maintains a hypercoagulable state [42, 61]. Although the mechanism of CHG index is not fully illustrated, the interactions among these mechanisms can be deeply understood in the future to provide new targets via CHG index for preventing CVD and improve prognosis.

### 4.3. Strengths and Limitations

Compared with previous studies, the present study has several notable advantages: [1] this is the first study utilizing prospective data exploring both CircS and CHG index on CVD jointly. Although prior research has examined CircS and CHG index independently, no study has assessed their combined impact on CVD. As a novel biomarker, the CHG index remains underexplored; the findings provide new insights into its role in CVD pathogenesis and its potential interaction with CircS. The use of prospective data also strengthens causal inference and expands the relevance of findings to time‐related clinical outcomes. [2] I used advanced assessment to estimate the interaction between CircS and CHG index. Significant additive and multiplicative interactions were detected in ELSA external cohort. [3] Beyond previous studies, the applied Fine–Gray competing risk model to account for noncardiovascular deaths, thereby minimizing potential confounding by competing events. Additionally, GEE models were used in sensitivity analyses to complement Cox regression, further reinforcing the robustness of findings. [4] RCS analyses were conducted to model the HR of CHG index, revealing a linear “dose–response” relationship. Identified cutoff points provide practical guidance for timely, targeted interventions aimed at improving CVD prognosis. [5] By comparing and incorporating CircS and CHG index with the well‐established SCORE2 Asia‐Pacific model, I demonstrated that the combination modestly enhances predictive performance, emphasizing the clinical relevance and performance of these indices in CVD risk stratification and management. [6] The incorporation of an external ELSA cohort addressed the robustness and rigidity of the findings from CHARLS study, which highlights the extension and utility of current findings.

This study also has limitations. First, although CHARLS provides a nationally representative sample, it may not fully capture CVD risk factors or population characteristics present in other regions or countries. Second, due to the observational design, residual confounding cannot be entirely excluded, and causal relationships are not definitively established. Third, outcomes were determined via self‐reported questionnaires, which might introduce reporting bias, and disease subtypes or common CVD biomarkers could not be distinguished for more granular analyses. Because disease identification in CHARLS was based on self‐reported data, which may lead to lower baseline discrimination power, the performance of the SCORE2 Asia‐Pacific model in this cohort was lower than expected and may therefore be underestimated compared with cohorts using medical examination data. Fourth, participants were dichotomized into high and low CHG index groups based on the median value; I tried the algorithm default optimal cutoff values and captured quite similar results like the median point, although the absence of standardized clinical level cut‐off points may limit the clinical interpretability of these thresholds, highlighting the need for more precise grouping criteria in future studies. Fifth, corresponding cost‐effectiveness analysis was not available. Cost‐effectiveness analysis can complement population‐based studies by estimating whether preventive or therapeutic strategies provide sufficient health benefits relative to their costs, therefore informing resource allocation and public health decision‐making. Due to the lack of detailed cost data, healthcare utilization records, and intervention‐specific economic parameters, I was unable to perform a cost‐effectiveness analysis in current study. Future studies may consider to incorporate economic evaluations to facilitate cost‐effectiveness analysis. Finally, the SCORE2 Asia‐Pacific model performed below expectations in the CHARLS cohort, potentially due to population heterogeneity, differing selection criteria, and lack of strict adherence to SCORE2‐recommended variables. Although alternative generalized models exit such as global Framingham model [62, 63], CHINA‐PAR risk model [64], and Japanese JALS risk score [65]. I specifically employed SCORE2 Asia‐Pacific model as Framingham model was generated earlier, and its updated version tends to overestimate CVD risk in Korea and produces heterogeneous risk estimates in Malaysia [62], although CHINA‐PAR and JALS are region‐specific and not widely endorsed by authoritative societies.

## 5. Conclusion

These results indicated that both the CHG index and CircS were positively associated with enhanced CVD risk, with the CHG index exhibiting a clear linear relationship. Significant additive or multiplicative interactions were observed between CircS and CHG index in ELSA study, their combined presence was associated with higher CVD risk compared with either factor alone. Furthermore, integrating CHG index and CircS into the SCORE2 Asia‐Pacific model modestly enhanced its predictive performance for long‐term CVD risk. This study may be regarded as the first clinical investigation of the joint association between CircS and CHG index, suggesting that combined evaluation could be regularly considered for CVD risk stratification and the optimization of primary prevention strategies.

**Nomenclature**
AP: attributable proportion
AUROC: area under the receiver operating characteristic curve
BIC: Bayesian information criterion
BMI: body mass index
CESD: Center for Epidemiologic Studies‐Depression scale
CHARLS: China Health and Retirement Longitudinal Study
CHG: cholesterol, high‐density lipoprotein and glucose
CI: confidence interval
CircS: circadian syndrome
CRP: C‐reactive protein
CVD: cardiovascular disease
DBP: diastolic blood pressure
DN: diabetic nephropathy
DR: diabetic retinopathy
ELSA: English Longitudinal Study of Ageing
ESC: European Society of Cardiology
FBG: fasting blood glucose
GEE: generalized estimating equation
GFR: glomerular filtration rate
GLM: generalized linear model
HbA1c: glycosylated hemoglobin A1c
HDL‐C: high‐density lipoprotein cholesterol
HGB: hemoglobin
HR: hazard ratio
IADL: instrumental activities of daily living
IQR: interquartile ranges
IR: insulin resistance
K–M curves: Kaplan–Meier curves
K–S test: Kolmogorov–Smirnov test
LAP: lipid accumulation product
LDL‐C: low‐density lipoprotein cholesterol
MetS: metabolic syndrome
MICE: multiple imputation by chained equations
NAFLD: nonalcoholic fatty liver disease
RCS: restricted cubic spline
RERI: relative excess risk due to interaction
ROC: receiver operating characteristic
RPF: renal plasma flow
SBP: systolic blood pressure
SCORE2: Systematic COronary Risk Evaluation 2
SD: standard deviations
sHR: subdistributon hazard ratio
SI: synergy index
STROBE: Strengthening the Reporting of Observational Studies in Epidemiology
T2D: Type 2 diabetes
TC: total cholesterol
TG: triglycerides
TyG: triglyceride–glucose
VAI: visceral adiposity index
WC: waist circumference

## Author Contributions

Concept and design, acquisition, analysis, or interpretation of data, drafting of the manuscript, critical revision of the manuscript for important intellectual content, and supervision: S.J.

## Funding

No funding was received for this manuscript.

## Disclosure

The corresponding author read and approved the final manuscript.

## Ethics Statement

All participants entered CHARLS cohorts and ELSA cohort have achieved ethical approval. Informed written consents were obtained from all participants.

## Consent

The corresponding author agrees with the publication.

## Conflicts of Interest

The author declares no conflicts of interests.

## Supporting Information

Additional supporting information can be found online in the Supporting Information section.

## Supporting information


**Supporting Information 1** Figure S1. Kaplan–Meier plots for cumulative CVD risk by different CHG index IQR (classified into Q1, Q2, Q3, and Q4). Abbreviations: CVD, cardiovascular; CHG, cholesterol, high‐density lipoprotein and glucose; and IQR, interquartile ranges.

**Supporting Information 2** Figure S2. The flow chart of participants selection in ELSA cohort.

**Supporting Information 3** Figure S3. Kaplan–Meier plots for cumulative CVD risk results in ELSA cohort. (A) Cumulative risk results by CircS. (B) Cumulative risk results by CHG index. (C) Cumulative risk results by CHG index (quartile). (D) Cumulative risk results by the combination of CircS and CHG index. Abbreviations: CVD, cardiovascular disease; CircS, circadian syndrome; CHG, cholesterol, high‐density lipoprotein and glucose.

**Supporting Information 4** Figure S4. RCS models for the relationship between CHG index and CVD risk. Abbreviations: RCS, restricted cubic spline; CHG, cholesterol, high‐density lipoprotein and glucose; CVD, cardiovascular disease; HR, hazard ratio; CI, confidence interval.

**Supporting Information 5** Table S1. Strengthening the reporting of observational studies in epidemiology checklist for this study.

**Supporting Information 6** Table S2. Sensitivity analysis results.

**Supporting Information 7** Table S3. Subgroup analysis results for the joint association of CircS and CHG index.

**Supporting Information 8** Table S4. Basic demographic characteristics of included participants in ELSA cohort.

**Supporting Information 9** Table S5. Cox regression model results for CircS and CHG index and their joint risk for CVD in ELSA cohort.

**Supporting Information 10** Table S6. Joint association and interactions between CircS and CHG on CVD in ELSA cohort.

## Data Availability

The datasets analyzed in the current study are public, specific data are available from the corresponding author on reasonable request.
