# Supplementary figures and images for: Joint Association of Cholesterol, High‐Density Lipoprotein and Glucose Index, and Circadian Syndrome With Incidence of Cardiovascular Disease: Results From National Longitudinal Prospective Studies
Source: Cardiovasc Ther. 2026 Jul 7;2026:1001613. doi: 10.1155/cdr/1001613 (PMC13341945; doi:10.1155/cdr/1001613)

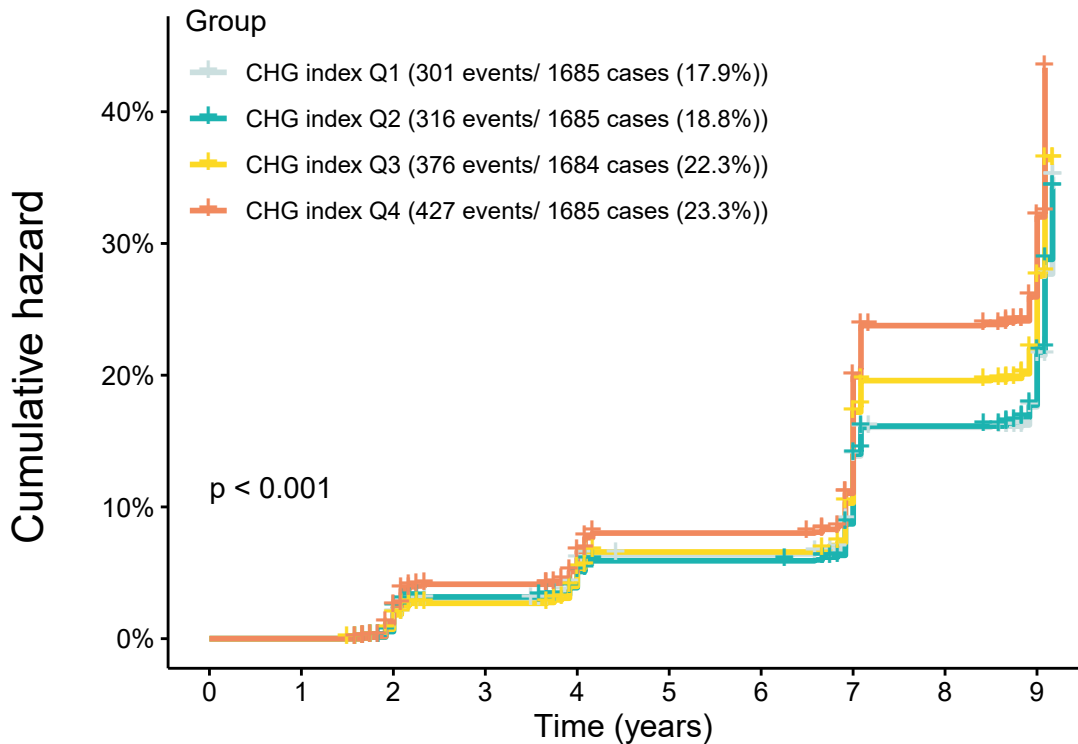

|              |      |      |      |      |      |      |      |      |      |     |
|--------------|------|------|------|------|------|------|------|------|------|-----|
| CHG_index Q1 | 1685 | 1685 | 1657 | 1575 | 1515 | 1415 | 1415 | 1355 | 1217 | 911 |
| CHG_index Q2 | 1685 | 1685 | 1664 | 1582 | 1550 | 1453 | 1453 | 1396 | 1240 | 961 |
| CHG_index Q3 | 1684 | 1684 | 1657 | 1574 | 1527 | 1432 | 1432 | 1364 | 1186 | 892 |
| CHG_index Q4 | 1685 | 1685 | 1650 | 1557 | 1519 | 1416 | 1416 | 1348 | 1117 | 832 |

Supplement: Supplementary file 1 — Supporting Information 1 Figure S1. Kaplan–Meier plots for cumulative CVD risk by different CHG index IQR (classified into Q1, Q2, Q3, and Q4). Abbreviations: CVD, cardiovascular; CHG, cholesterol, high‐density lipoprotein and glucose; and IQR, interquartile ranges. [file CDR-2026-1001613-s009.pdf]

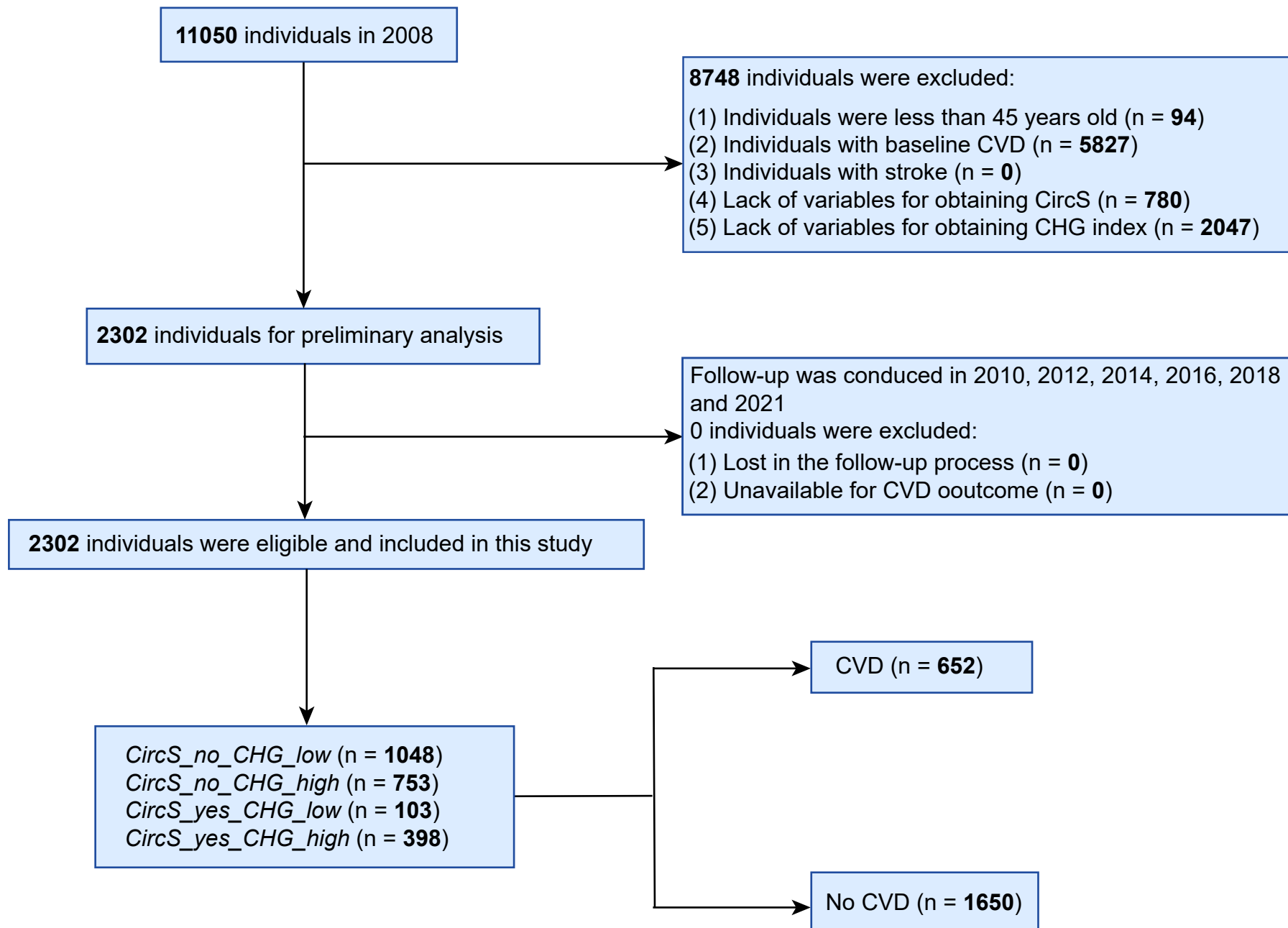

Supplement: Supplementary file 2 — Supporting Information 2 Figure S2. The flow chart of participants selection in ELSA cohort. [file CDR-2026-1001613-s001.pdf]

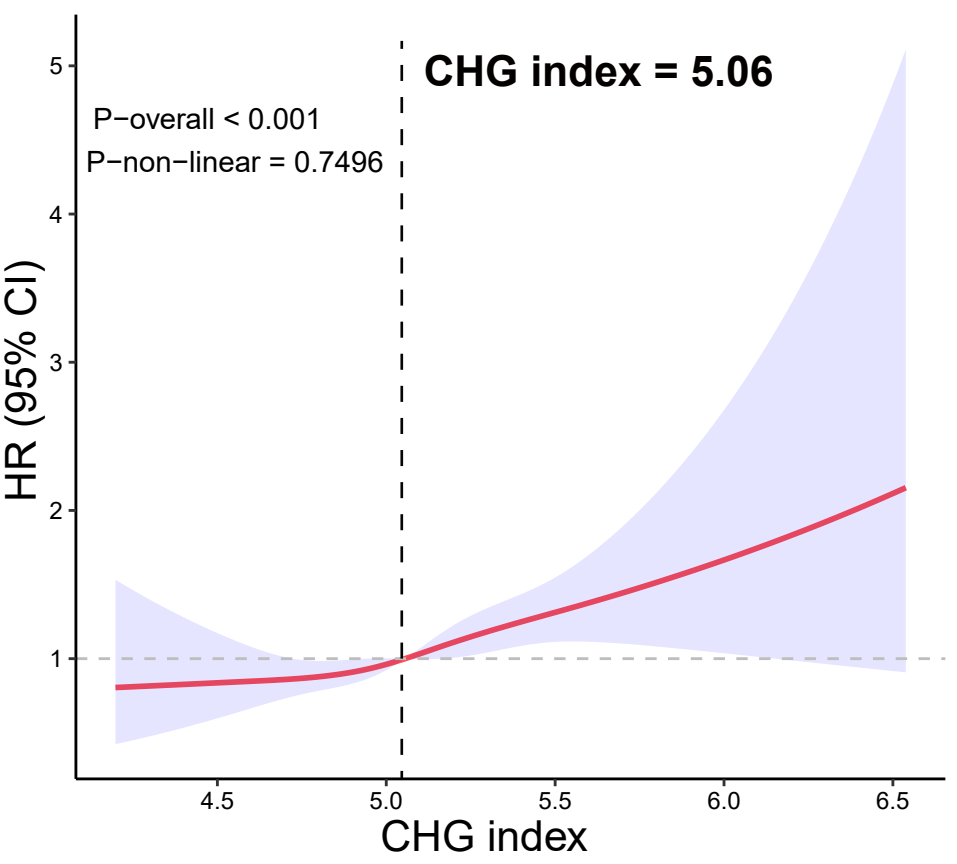

Supplement: Supplementary file 4 — Supporting Information 4 Figure S4. RCS models for the relationship between CHG index and CVD risk. Abbreviations: RCS, restricted cubic spline; CHG, cholesterol, high‐density lipoprotein and glucose; CVD, cardiovascular disease; HR, hazard ratio; CI, confidence interval. [file CDR-2026-1001613-s003.pdf]
