# Supplementary material for: Joint Association of Cholesterol, High‐Density Lipoprotein and Glucose Index, and Circadian Syndrome With Incidence of Cardiovascular Disease: Results From National Longitudinal Prospective Studies
Source: Cardiovasc Ther. 2026 Jul 7;2026:1001613. doi: 10.1155/cdr/1001613 (PMC13341945; doi:10.1155/cdr/1001613)
Supplement: Supplementary file 3 — Supporting Information 3 Figure S3. Kaplan–Meier plots for cumulative CVD risk results in ELSA cohort. (A) Cumulative risk results by CircS. (B) Cumulative risk results by CHG index. (C) Cumulative risk results by CHG index (quartile). (D) Cumulative risk results by the combination of CircS and CHG index. Abbreviations: CVD, cardiovascular disease; CircS, circadian syndrome; CHG, cholesterol, high‐density lipoprotein and glucose. [file CDR-2026-1001613-s002.pdf]

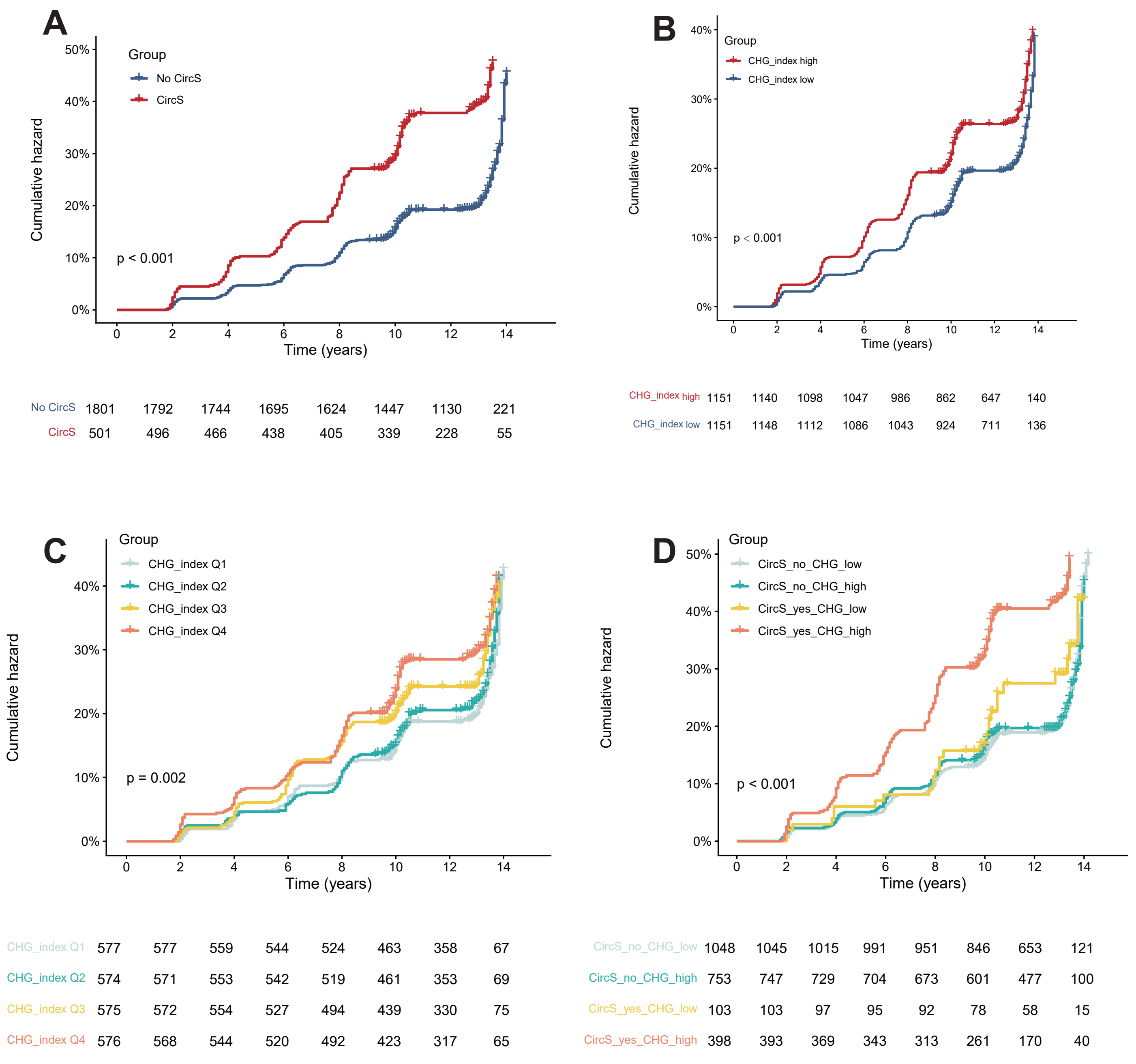
A
Cumulative hazard
Group
No CircS
CircS
p < 0.001
Time (years)
No CircS 1801 1792 1744 1695 1624 1447 1130 221
CircS 501 496 466 438 405 339 228 55
B
Cumulative hazard
Group
CHG_index high
CHG_index low
p < 0.001
Time (years)
CHG_index high 1151 1140 1098 1047 986 862 647 140
CHG_index low 1151 1148 1112 1086 1043 924 711 136
C
Cumulative hazard
Group
CHG_index Q1
CHG_index Q2
CHG_index Q3
CHG_index Q4
p = 0.002
Time (years)
CHG_index Q1 577 577 559 544 524 463 358 67
CHG_index Q2 574 571 553 542 519 461 353 69
CHG_index Q3 575 572 554 527 494 439 330 75
CHG_index Q4 576 568 544 520 492 423 317 65
D
Cumulative hazard
Group
CircS_no_CHG_low
CircS_no_CHG_high
CircS_yes_CHG_low
CircS_yes_CHG_high
p < 0.001
Time (years)
CircS_no_CHG_low 1048 1045 1015 991 951 846 653 121
CircS_no_CHG_high 753 747 729 704 673 601 477 100
CircS_yes_CHG_low 103 103 97 95 92 78 58 15
CircS_yes_CHG_high 398 393 369 343 313 261 170 40
