# Supplementary material for: Joint Association of Cholesterol, High‐Density Lipoprotein and Glucose Index, and Circadian Syndrome With Incidence of Cardiovascular Disease: Results From National Longitudinal Prospective Studies
Source: Cardiovasc Ther. 2026 Jul 7;2026:1001613. doi: 10.1155/cdr/1001613 (PMC13341945; doi:10.1155/cdr/1001613)
Supplement: Supplementary file 6 — Supporting Information 6 Table S2. Sensitivity analysis results. [file CDR-2026-1001613-s005.docx]

**Table S2**. Sensitivity analysis results

| **Variables** | **Case** | **Unadjusted model** | |  | **Model 1** | |  | **Model 2** | |  | **Model 3** | |  |
| --- | --- | --- | --- | --- | --- | --- | --- | --- | --- | --- | --- | --- | --- |
|  |  | HR (95% CI) | P value |  | HR (95% CI) | P value |  | HR (95% CI) | P value |  | HR (95% CI) | P value |  |
| **No memory or psychiatric disorders** |  |  |  |  |  |  |  |  |  |  |  |  |  |
| CircS and CHG index |  |  |  |  |  |  |  |  |  |  |  |  |  |
| CircS_no_CHG_low | 3107 | 1 (Ref.) |  |  | 1 (Ref.) |  |  | 1 (Ref.) |  |  | 1 (Ref.) |  |  |
| CircS_no_CHG_high | 1975 | 1.25 (1.10-1.42) | < 0.001 |  | 1.27 (1.11-1.44) | < 0.001 |  | 1.26 (1.10-1.43) | < 0.001 |  | 1.23 (1.08-1.39) | 0.002 |  |
| CircS_yes_CHG_low | 263 | 1.60 (1.23-2.08) | < 0.001 |  | 1.47 (1.13-1.91) | 0.004 |  | 1.48 (1.14-1.93) | 0.003 |  | 1.16 (0.89-1.52) | 0.265 |  |
| CircS_yes_CHG_high | 1394 | 1.67 (1.47-1.91) | < 0.001 |  | 1.62 (1.42-1.85) | < 0.001 |  | 1.63 (1.42-1.86) | < 0.001 |  | 1.26 (1.09-1.45) | 0.002 |  |
|  |  |  |  |  |  |  |  |  |  |  |  |  |  |
| **No cognitive disease** |  |  |  |  |  |  |  |  |  |  |  |  |  |
| CircS and CHG index |  |  |  |  |  |  |  |  |  |  |  |  |  |
| CircS_no_CHG_low | 3107 | 1 (Ref.) |  |  | 1 (Ref.) |  |  | 1 (Ref.) |  |  | 1 (Ref.) |  |  |
| CircS_no_CHG_high | 1975 | 1.32 (1.14-1.52) | < 0.001 |  | 1.34 (1.16-1.54) | < 0.001 |  | 1.33 (1.15-1.53) | < 0.001 |  | 1.29 (1.11-1.49) | < 0.001 |  |
| CircS_yes_CHG_low | 263 | 1.55 (1.14-2.11) | 0.005 |  | 1.45 (1.06-1.97) | 0.019 |  | 1.46 (1.07-1.99) | 0.016 |  | 1.11 (0.81-1.53) | 0.501 |  |
| CircS_yes_CHG_high | 1394 | 1.71 (1.47-1.99) | < 0.001 |  | 1.66 (1.42-1.93) | < 0.001 |  | 1.66 (1.42-1.94) | < 0.001 |  | 1.26 (1.07-1.49) | 0.006 |  |
|  |  |  |  |  |  |  |  |  |  |  |  |  |  |
| **GEE model** |  |  |  |  |  |  |  |  |  |  |  |  |  |
| CircS and CHG index |  | OR (95% CI) | P value |  | OR (95% CI) | P value |  | OR (95% CI) | P value |  | OR (95% CI) | P value |  |
| CircS_no_CHG_low | 3107 | 1 (Ref.) |  |  | 1 (Ref.) |  |  | 1 (Ref.) |  |  | 1 (Ref.) |  |  |
| CircS_no_CHG_high | 1975 | 1.27 (1.11-1.47) | < 0.001 |  | 1.29 (1.12-1.48) | < 0.001 |  | 1.28 (1.11-1.48) | < 0.001 |  | 1.25 (1.08-1.45) | 0.002 |  |
| CircS_yes_CHG_low | 263 | 1.62 (1.21-2.17) | 0.001 |  | 1.52 (1.13-2.05) | 0.005 |  | 1.54 (1.15-2.07) | 0.004 |  | 1.20 (0.88-1.63) | 0.249 |  |
| CircS_yes_CHG_high | 1394 | 1.74 (1.50-2.02) | < 0.001 |  | 1.69 (1.45-1.97) | < 0.001 |  | 1.70 (1.46-1.98) | < 0.001 |  | 1.30 (1.10-1.53) | 0.002 |  |
| **Abbreviations**: HR, hazard ratio; CircS, circadian syndrome; CHG index, cholesterol, high-density lipoprotein and glucose (CHG) index; OR, odd ratio; 95% CI, 95% confidence interval. Model 1: adjusted for age and gender; Model 2: adjusted for model 1 plus smoking, drinking, marriage, education, residential area and normal physical activity; Model 3: adjusted for model 2 plus diabetes, hypertension, dyslipidemia, kidney disease, liver disease and C-reactive protein. | | | | | | | | | | | | |  |
|  |  |  |  |  |  |  |  |  |  |  |  |  |  |
