# Supplementary material for: Joint Association of Cholesterol, High‐Density Lipoprotein and Glucose Index, and Circadian Syndrome With Incidence of Cardiovascular Disease: Results From National Longitudinal Prospective Studies
Source: Cardiovasc Ther. 2026 Jul 7;2026:1001613. doi: 10.1155/cdr/1001613 (PMC13341945; doi:10.1155/cdr/1001613)
Supplement: Supplementary file 7 — Supporting Information 7 Table S3. Subgroup analysis results for the joint association of CircS and CHG index. [file CDR-2026-1001613-s006.docx]

**Table S3**. Subgroup analysis results for the joint association of CircS and CHG index

|  | Case | CircS_no_CHG_low | CircS_no_CHG_high | CircS_yes_CHG_low | CircS_yes_CHG_high | P for interaction |  |
| --- | --- | --- | --- | --- | --- | --- | --- |
| Overall | 6739 | 1 (Ref.) | 1.26 (1.11-1.43) | 1.65 (1.28-2.12) | 1.67 (1.46-1.90) |  |  |
| Age (year) |  |  |  |  |  | 0.721 |  |
| 45-60 | 3866 | 1 (Ref.) | 1.22 (1.02-1.45) | 1.63 (1.11-2.41) | 1.60 (1.33-1.93) |  |  |
| 60-70 | 1907 | 1 (Ref.) | 1.46 (1.16-1.82) | 1.91 (1.28-2.85) | 1.73 (1.38-2.17) |  |  |
| 70-80 | 812 | 1 (Ref.) | 1.18 (0.84-1.65) | 0.96 (0.50-1.85) | 1.63 (1.15-2.29) |  |  |
| >=80 | 154 | 1 (Ref.) | 0.63 (0.18-2.24) | 1.26 (0.28-5.70) | 1.53 (0.62-3.75) |  |  |
| Gender |  |  |  |  |  | 0.665 |  |
| Female | 3543 | 1 (Ref.) | 1.26 (1.06-1.50) | 1.84 (1.34-2.52) | 1.62 (1.36-1.92) |  |  |
| Male | 3196 | 1 (Ref.) | 1.26 (1.05-1.52) | 1.39 (0.91-2.11) | 1.72 (1.4-2.10) |  |  |
| Smoking |  |  |  |  |  |  |  |
| No | 4630 | 1 (Ref.) | 1.30 (1.11-1.51) | 1.89 (1.42-2.52) | 1.67 (1.43-1.95) | 0.360 |  |
| Yes | 2109 | 1 (Ref.) | 1.17 (0.93-1.47) | 1.14 (0.68-1.94) | 1.64 (1.28-2.11) |  |  |
| Drinking |  |  |  |  |  | 0.320 |  |
| No | 4410 | 1 (Ref.) | 1.32 (1.13-1.54) | 1.88 (1.40-2.52) | 1.75 (1.50-2.05) |  |  |
| Yes | 2329 | 1 (Ref.) | 1.15 (0.92-1.43) | 1.21 (0.74-1.98) | 1.46 (1.15-1.85) |  |  |
| Residential area |  |  |  |  |  | 0.795 |  |
| Rural | 4428 | 1 (Ref.) | 1.22 (1.04-1.42) | 1.55 (1.16-2.09) | 1.66 (1.42-1.96) |  |  |
| Urban | 2311 | 1 (Ref.) | 1.35 (1.08-1.68) | 1.95 (1.20-3.19) | 1.70 (1.35-2.13) |  |  |
| Diabetes |  |  |  |  |  | 0.721 |  |
| No | 6387 | 1 (Ref.) | 1.23 (1.08-1.40) | 1.62 (1.25-2.10) | 1.56 (1.36-1.79) |  |  |
| Yes | 352 | 1 (Ref.) | 1.62 (0.72-3.67) | 2.11 (0.67-6.64) | 2.34 (1.07-5.11) |  |  |
| Hypertension |  |  |  |  |  | 0.085 |  |
| No | 5255 | 1 (Ref.) | 1.23 (1.06-1.42) | 1.61 (1.15-2.26) | 1.57 (1.32-1.87) |  |  |
| Yes | 1484 | 1 (Ref.) | 1.25 (0.97-1.61) | 1.14 (0.77-1.68) | 1.17 (0.94-1.46) |  |  |
| Dyslipidemia |  |  |  |  |  | 0.172 |  |
| No | 6195 | 1 (Ref.) | 1.27 (1.11-1.45) | 1.64 (1.24-2.19) | 1.55 (1.34-1.80) |  |  |
| Yes | 544 | 1 (Ref.) | 0.87 (0.55-1.37) | 0.84 (0.47-1.51) | 1.09 (0.75-1.59) |  |  |
| Kidney disease |  |  |  |  |  | 0.707 |  |
| No | 6418 | 1 (Ref.) | 1.25 (1.10-1.43) | 1.59 (1.22-2.07) | 1.65 (1.44-1.89) |  |  |
| Yes | 321 | 1 (Ref.) | 1.46 (0.83-2.56) | 2.73 (1.14-6.54) | 1.88 (1.12-3.17) |  |  |
| BMI group |  |  |  |  |  | 0.880 |  |
| Low BMI | 3370 | 1 (Ref.) | 1.18 (0.97-1.43) | 1.62 (1.13-2.32) | 1.67 (1.34-2.09) |  |  |
| High BMI | 3369 | 1 (Ref.) | 1.22 (1.02-1.46) | 1.61 (1.13-2.30) | 1.53 (1.28-1.82) |  |  |
| CRP group |  |  |  |  |  | 0.231 |  |
| Low CRP | 3383 | 1 (Ref.) | 1.13 (0.93-1.37) | 1.66 (1.16-2.38) | 1.77 (1.44-2.17) |  |  |
| High CRP | 3356 | 1 (Ref.) | 1.27 (1.06-1.50) | 1.58 (1.11-2.24) | 1.46 (1.23-1.74) |  |  |
| Estimate risks were presented in HR (95% CI).  Abbreviations: HR, hazard ratio; 95% CI, 95% confidnce interval; CircS: circadian syndrome; CHG index, cholesterol, high-density lipoprotein and glucose (CHG) index; BMI, body mass index; CRP, C-reactive protein. | | | | | | |  |
|  |  |  |  |  |  |  |  |
