# Supplementary material for: Joint Association of Cholesterol, High‐Density Lipoprotein and Glucose Index, and Circadian Syndrome With Incidence of Cardiovascular Disease: Results From National Longitudinal Prospective Studies
Source: Cardiovasc Ther. 2026 Jul 7;2026:1001613. doi: 10.1155/cdr/1001613 (PMC13341945; doi:10.1155/cdr/1001613)
Supplement: Supplementary file 8 — Supporting Information 8 Table S4. Basic demographic characteristics of included participants in ELSA cohort. [file CDR-2026-1001613-s007.docx]

**Table S4.** Basic demographic characteristics of included participants in ELSA cohort

| **Characteristic** | **Overall (N = 2302) (N (%)/mean ± SD/median (Q1, Q3))** | **No CVD (N = 1650) (N (%)/mean ± SD/median (Q1, Q3))** | **CVD (N = 652) (N (%)/mean ± SD/median (Q1, Q3))** | **P value** |  |
| --- | --- | --- | --- | --- | --- |
| **CircS** |  |  |  | <0.001 |  |
| **No** | 1,801 (78%) | 1,344 (81%) | 457 (70%) |  |  |
| **Yes** | 501 (22%) | 306 (19%) | 195 (30%) |  |  |
| **CHG_index** | 5.07 ± 0.30 | 5.05 ± 0.29 | 5.11 ± 0.30 | <0.001 |  |
| **CHG index median** |  |  |  | <0.001 |  |
| **CHG high** | 1,151 (50%) | 787 (48%) | 364 (56%) |  |  |
| **CHG low** | 1,151 (50%) | 863 (52%) | 288 (44%) |  |  |
| **CHG index quantile** |  |  |  | 0.002 |  |
| **Q1** | 577 (25%) | 440 (27%) | 137 (21%) |  |  |
| **Q2** | 574 (25%) | 423 (26%) | 151 (23%) |  |  |
| **Q3** | 575 (25%) | 404 (24%) | 171 (26%) |  |  |
| **Q4** | 576 (25%) | 383 (23%) | 193 (30%) |  |  |
| **CircS_CHG_index** |  |  |  | <0.001 |  |
| ***CircS_no_CHG_low*** | 1,048 (46%) | 789 (48%) | 259 (40%) |  |  |
| ***CircS_no_CHG_high*** | 753 (33%) | 555 (34%) | 198 (30%) |  |  |
| ***CircS_yes_CHG_high*** | 103 (4.5%) | 74 (4.5%) | 29 (4.4%) |  |  |
| ***CircS_yes_CHG_low*** | 398 (17%) | 232 (14%) | 166 (25%) |  |  |
| **Age** | 61.00 (57.00, 67.00) | 61.00 (56.00, 66.00) | 63.00 (59.00, 70.00) | <0.001 |  |
| **Gender** |  |  |  | <0.001 |  |
| **Female** | 1,006 (44%) | 680 (41%) | 326 (50%) |  |  |
| **Male** | 1,296 (56%) | 970 (59%) | 326 (50%) |  |  |
| **Standardized education level** |  |  |  | 0.84 |  |
| **Less than lower secondary education** | 562 (24%) | 404 (24%) | 158 (24%) |  |  |
| **Tertiary education** | 532 (23%) | 376 (23%) | 156 (24%) |  |  |
| **Upper secondary education** | 1,208 (52%) | 870 (53%) | 338 (52%) |  |  |
| **Marriage** |  |  |  | 0.26 |  |
| **Divorced** | 274 (12%) | 197 (12%) | 77 (12%) |  |  |
| **Married** | 1,639 (71%) | 1,187 (72%) | 452 (69%) |  |  |
| **Never married** | 148 (6.4%) | 105 (6.4%) | 43 (6.6%) |  |  |
| **Separated** | 24 (1.0%) | 19 (1.2%) | 5 (0.8%) |  |  |
| **Widowed** | 217 (9.4%) | 142 (8.6%) | 75 (12%) |  |  |
| **Hypertension (yes)** | 673 (29%) | 420 (25%) | 253 (39%) | <0.001 |  |
| **Diabetes (yes)** | 65 (2.8%) | 39 (2.4%) | 26 (4.0%) | 0.034 |  |
| **Cancer (yes)** | 136 (5.9%) | 93 (5.6%) | 43 (6.6%) | 0.38 |  |
| **Pulmonary disease (yes)** | 62 (2.7%) | 39 (2.4%) | 23 (3.5%) | 0.12 |  |
| **Psychiatric disorder (yes)** | 177 (7.7%) | 111 (6.7%) | 66 (10%) | 0.006 |  |
| **Arthritis (yes)** | 697 (30%) | 447 (27%) | 250 (38%) | <0.001 |  |
| **Asthma (yes)** | 263 (11%) | 167 (10%) | 96 (15%) | 0.002 |  |
| **High cholesterol (yes)** | 649 (28%) | 424 (26%) | 225 (35%) | <0.001 |  |
| **Angina (yes)** | 20 (0.9%) | 5 (0.3%) | 15 (2.3%) | <0.001 |  |
| **Arrhythmia (yes)** | 11 (0.5%) | 4 (0.2%) | 7 (1.1%) | 0.015 |  |
| **Osteoporosis (yes)** | 92 (4.0%) | 56 (3.4%) | 36 (5.5%) | 0.019 |  |
| **Anti hypertension drug use (yes)** | 497 (22%) | 302 (18%) | 195 (30%) | <0.001 |  |
| **Anti diabetes drug use (yes)** | 47 (2.0%) | 22 (1.3%) | 25 (3.8%) | <0.001 |  |
| **Anti pulmonary disease drug use (yes)** | 16 (0.7%) | 9 (0.5%) | 7 (1.1%) | 0.17 |  |
| **Anti asthma drug use (yes)** | 174 (7.6%) | 109 (6.6%) | 65 (10.0%) | 0.006 |  |
| **Cancer treatment (yes)** | 46 (2.0%) | 32 (1.9%) | 14 (2.1%) | 0.75 |  |
| **Anti high cholesterol drug use (yes)** | 360 (16%) | 227 (14%) | 133 (20%) | <0.001 |  |
| **Dementia (yes)** | 3 (0.1%) | 2 (0.1%) | 1 (0.2%) | >0.99 |  |
| **Memory disorders (yes)** | 3 (0.1%) | 2 (0.1%) | 1 (0.2%) | >0.99 |  |
| **Pain (yes)** | 802 (35%) | 516 (31%) | 286 (44%) | <0.001 |  |
| **Alcohol use (yes)** | 2,129 (92%) | 1,528 (93%) | 601 (92%) | 0.73 |  |
| **Smoking (yes)** | 1,236 (54%) | 857 (52%) | 379 (58%) | 0.007 |  |
| **Weight, kg** | 76.65 (67.30, 87.20) | 75.20 (66.40, 85.80) | 79.95 (68.95, 89.45) | <0.001 |  |
| **CES-D** | 0.00 (0.00, 1.00) | 0.00 (0.00, 1.00) | 0.00 (0.00, 2.00) | 0.024 |  |
| **Consistent shortness breath (yes)** | 566 (25%) | 357 (22%) | 209 (32%) | <0.001 |  |
| **DBP, mmhg** | 76.00 (69.50, 82.50) | 75.50 (69.50, 82.50) | 76.50 (69.50, 83.00) | 0.14 |  |
| **SBP, mmhg** | 130.00 (120.50, 141.50) | 129.00 (120.00, 140.50) | 132.50 (121.50, 143.50) | <0.001 |  |
| **Pulse** | 65.00 (59.00, 71.00) | 65.00 (59.00, 71.45) | 64.50 (58.50, 71.00) | 0.64 |  |
| **BMI, kg/m2** | 27.20 (24.70, 30.50) | 26.90 (24.40, 30.20) | 27.90 (25.50, 31.30) | <0.001 |  |
| **Height, m** | 1.66 (1.60, 1.74) | 1.66 (1.60, 1.73) | 1.67 (1.61, 1.74) | 0.006 |  |
| **Waist, cm** | 95.63 ± 13.18 | 94.62 ± 13.00 | 98.19 ± 13.28 | <0.001 |  |
| **Waist hip ratio** | 0.89 (0.83, 0.95) | 0.88 (0.82, 0.94) | 0.90 (0.85, 0.95) | <0.001 |  |
| **Respiratory tract infection (yes)** | 189 (8.2%) | 112 (6.8%) | 77 (12%) | <0.001 |  |
| **Anti depression drug use (yes)** | 56 (2.4%) | 34 (2.1%) | 22 (3.4%) | 0.065 |  |
| **Abbreviations**: SD, standard derivation; CVD, cardiovascular disease; CircS: circadian syndrome; CHG index, cholesterol, high-density lipoprotein and glucose (CHG) index; CESD, 8-item Center for Epidemiologic Studies Depression Scale; DBP, diastolic blood pressure; SBP, systolic blood pressure; BMI, body mass index. | | | | |  |
|  |  |  |  |  |  |
|  |  |  |  |  |  |
