# Supplementary material for: Joint Association of Cholesterol, High‐Density Lipoprotein and Glucose Index, and Circadian Syndrome With Incidence of Cardiovascular Disease: Results From National Longitudinal Prospective Studies
Source: Cardiovasc Ther. 2026 Jul 7;2026:1001613. doi: 10.1155/cdr/1001613 (PMC13341945; doi:10.1155/cdr/1001613)
Supplement: Supplementary file 9 — Supporting Information 9 Table S5. Cox regression model results for CircS and CHG index and their joint risk for CVD in ELSA cohort. [file CDR-2026-1001613-s008.docx]

**Table S5**. Cox regression model results for CircS and CHG index and their joint risk for CVD in ELSA cohort

| **Variables** | **Case** | **Unadjusted model** | |  | **Model 1** | |  | **Model 2** | |  | **Model 3** | |  |
| --- | --- | --- | --- | --- | --- | --- | --- | --- | --- | --- | --- | --- | --- |
|  |  | **HR (95% CI)** | **P value** |  | **HR (95% CI)** | **P value** |  | **HR (95% CI)** | **P value** |  | **HR (95% CI)** | **P value** |  |
| **CircS** |  |  |  |  |  |  |  |  |  |  |  |  |  |
| **No** | 1801 | 1 (Ref.) |  |  | 1 (Ref.) |  |  | 1 (Ref.) |  |  | 1 (Ref.) |  |  |
| **Yes** | 501 | 1.78 (1.51-2.11) | < 0.001 |  | 1.63 (1.38-1.93) | < 0.001 |  | 1.64 (1.38-1.95) | < 0.001 |  | 1.31 (1.08-1.59) | 0.005 |  |
| **CHG index (continuous)** |  | 1.69 (1.30-2.18) | < 0.001 |  | 1.56 (1.20-2.02) | < 0.001 |  | 1.53 (1.18-2.00) | 0.002 |  | 1.25 (0.95-1.65) | 0.107 |  |
| **CHG index (median)** |  |  |  |  |  |  |  |  |  |  |  |  |  |
| **Low** | 1151 | 1 (Ref.) |  |  | 1 (Ref.) |  |  | 1 (Ref.) |  |  | 1 (Ref.) |  |  |
| **High** | 1151 | 1.31 (1.13-1.53) | < 0.001 |  | 1.23 (1.05-1.44) | 0.009 |  | 1.22 (1.04-1.43) | 0.013 |  | 1.11 (0.94-1.30) | 0.225 |  |
| **CHG index (quartile)** |  |  |  |  |  |  |  |  |  |  |  |  |  |
| **Q1** | 577 | 1 (Ref.) |  |  | 1 (Ref.) |  |  | 1 (Ref.) |  |  | 1 (Ref.) |  |  |
| **Q2** | 574 | 1.09 (0.86-1.37) | 0.474 |  | 1.04 (0.83-1.31) | 0.733 |  | 1.03 (0.82-1.30) | 0.773 |  | 0.99 (0.79-1.26) | 0.962 |  |
| **Q3** | 575 | 1.28 (1.02-1.60) | 0.032 |  | 1.17 (0.93-1.47) | 0.181 |  | 1.15 (0.92-1.45) | 0.219 |  | 1.05 (0.84-1.33) | 0.658 |  |
| **Q4** | 576 | 1.47 (1.18-1.83) | < 0.001 |  | 1.36 (1.09-1.70) | 0.007 |  | 1.34 (1.07-1.67) | 0.011 |  | 1.16 (0.92-1.46) | 0.219 |  |
| **CircS and CHG index** |  |  |  |  |  |  |  |  |  |  |  |  |  |
| **CircS_no_CHG_low** | 1048 | 1 (Ref.) |  |  | 1 (Ref.) |  |  | 1 (Ref.) |  |  | 1 (Ref.) |  |  |
| **CircS_no_CHG_high** | 753 | 1.04 (0.87-1.25) | 0.663 |  | 0.98 (0.81-1.18) | 0.832 |  | 0.97 (0.80-1.17) | 0.761 |  | 0.94 (0.78-1.14) | 0.521 |  |
| **CircS_yes_CHG_low** | 103 | 1.18 (0.80-1.73) | 0.403 |  | 1.02 (0.69-1.50) | 0.933 |  | 1.02 (0.69-1.51) | 0.908 |  | 0.84 (0.57-1.25) | 0.399 |  |
| **CircS_yes_CHG_high** | 398 | 2.01 (1.65-2.44) | < 0.001 |  | 1.80 (1.48-2.19) | < 0.001 |  | 1.80 (1.47-2.20) | < 0.001 |  | 1.41 (1.13-1.76) | 0.003 |  |
| **Abbreviations**: HR, hazard ratio; 95% CI, 95% confidence interval; CircS, circadian syndrome; CHG index, cholesterol, high-density lipoprotein and glucose (CHG) index.  **Model 1**: adjusted for age and gender;  **Model 2**: adjusted for model 1 plus smoking, drinking, marriage and education level;  **Model 3**: adjusted for model 2 plus diabetes, hypertension, high cholesterol, pulmonary disease, cancer and BMI. | | | | | | | | | | | | |  |
|  |  |  |  |  |  |  |  |  |  |  |  |  |  |
