# Supplementary material for: Joint Association of Cholesterol, High‐Density Lipoprotein and Glucose Index, and Circadian Syndrome With Incidence of Cardiovascular Disease: Results From National Longitudinal Prospective Studies
Source: Cardiovasc Ther. 2026 Jul 7;2026:1001613. doi: 10.1155/cdr/1001613 (PMC13341945; doi:10.1155/cdr/1001613)
Supplement: Supplementary file 10 — Supporting Information 10 Table S6. Joint association and interactions between CircS and CHG on CVD in ELSA cohort. [file CDR-2026-1001613-s004.docx]

**Table S6.** Joint association and interactions between CircS and CHG on CVD in ELSA cohort

| Interactive indices | Interactive effects (95% CI) | |
| --- | --- | --- |
|  | In all populations (95% CI) | P value |
| Additive effect |  |  |
| RERI | 0.79 (0.24, 1.33) | 0.002 |
| AP | 0.39 (0.14, 0.64) | 0.001 |
| SI | 4.58 (0.46, 45.80) | 0.098 |
| Multiplicative scale | 1.64 (1.06, 2.53) | 0.027 |
| **Abbreviation**: CI, confidence interval; RERI, relative excess risk due to interaction; AP, proportion attributable to interaction; SI, synergy index. | | |
